# Richards’s curve induced Banach space valued multivariate neural network approximation

**DOI:** 10.1007/s40065-022-00414-9

**Published:** 2022-12-13

**Authors:** George A. Anastassiou, Seda Karateke

**Affiliations:** 1 Department of Mathematical Sciences, University of Memphis, Memphis, TN 38152 USA; 2 Department of Computer Engineering, Faculty of Engineering, Istanbul Topkapı University, Zeytinburnu, 34087 Istanbul, Turkey

**Keywords:** 41A17, 41A25, 41A30, 41A36

## Abstract

Here, we present multivariate quantitative approximations of Banach space valued continuous multivariate functions on a box or ${\mathbb {R}}^{N},$ $N\in {\mathbb {N}},$ by the multivariate normalized, quasi-interpolation, Kantorovich-type and quadrature-type neural network operators. We examine also the case of approximation by iterated operators of the last four types. These approximations are achieved by establishing multidimensional Jackson type inequalities involving the multivariate modulus of continuity of the engaged function or its high-order Fréchet derivatives. Our multivariate operators are defined using a multidimensional density function induced by the Richards’s curve, which is a generalized logistic function. The approximations are pointwise, uniform and $L_{p}.$ The related feed-forward neural network is with one hidden layer.

## Introduction

Anastassiou in [2, 3], see chapters 2–5, was the first to establish neural network approximations to continuous functions with rates by very specifically defined neural network operators of Cardaliaguet–Euvrard and “Squashing” types, by employing the modulus of continuity of the engaged function or its high-order derivative, and producing very tight Jackson type inequalities. He treats there both the univariate and multivariate cases. The defining these operators “bell-shaped” and “squashing” functions are assumed to be of compact support. Also, in [3], he gives the *N*th-order asymptotic expansion for the error of weak approximation of these two operators to a special natural class of smooth functions; see chapters 4–5 there.

Motivations for this work are the article [22] of Chen and Cao, and [4–20, 23, 24].

Here, we perform multivariate sigmoid function by Richards’s curve [30] based neural network approximations to continuous functions over boxes or over the whole $${\mathbb {R}}^{N},$$ $$N\in {\mathbb {N}},$$ and also iterated and $$ L_{p}$$ approximations. All convergences here are with rates expressed via the multivariate modulus of continuity of the involved function or its high-order Fréchet derivative and given by very tight multidimensional Jackson type inequalities.

We come up with the “right” precisely defined multivariate normalized, quasi-interpolation neural network operators related to boxes or ${\mathbb {R}}^{N},$ as well as Kantorovich-type and quadrature-type-related operators on ${\mathbb {R}}^{N}.$ Our boxes are not necessarily symmetric to the origin. In preparation to prove our results, we establish important properties of the basic multivariate density function induced by the sigmoid function related to Richards’s curve and defining our operators. Richards’s curve among others has been used for modeling COVID-19 infection trajectory [26].

Feed-forward neural networks (FNNs) with one hidden layer, the only type of networks we deal with in this article, are mathematically expressed as

$$\begin{aligned} N_{n}\left( x\right) =\sum _{j=0}^{n}c_{j}\sigma \left( \left\langle a_{j}\cdot x\right\rangle +b_{j}\right) ,\quad x\in {\mathbb {R}}^{s},\ s\in {\mathbb {N}}, \end{aligned}$$

where for $0\le j\le n,$ $b_{j}\in {\mathbb {R}}$ are the thresholds, $a_{j}\in {\mathbb {R}}^{s}$ are the connection weights, $c_{j}\in {\mathbb {R}}$ are the coefficients, $\left\langle a_{j}\cdot x\right\rangle$ is the inner product of $a_{j}$ and *x*,  and $\sigma$ is the activation function of the network. In many fundamental network models, the activation function is based on the Richards’s curve sigmoid function. About neural networks, see [25, 27, 28].

## Background

A Richards’s curve is [30]

1 $$\begin{aligned} \varphi \left( x\right) =\frac{1}{1+{\textrm{e}}^{-\mu x}};\quad x\in {\mathbb {R}} , \mu >0, \end{aligned}$$

which is strictly increasing on ${\mathbb {R}},$ and it is a sigmoid function. For small $0<\mu <1,$ our Richards’s curve, which is a smooth function, is expected to behave better than the ReLu activation function. We have that $\varphi \left( +\infty \right) =1$ and $\varphi \left( -\infty \right) =0.$

We consider the following activation function:

2 $$\begin{aligned} G\left( x\right) =\frac{1}{2}\left( \varphi \left( x+1\right) -\varphi \left( x-1\right) \right) ,\quad x\in {\mathbb {R}}, \end{aligned}$$

which is $G\left( x\right) >0,$ all $x\in {\mathbb {R}}.$

We have that

3 $$\begin{aligned} \varphi \left( 0\right) =\frac{1}{2}\quad \text {and}\quad \varphi \left( x\right) =1-\varphi \left( -x\right) . \end{aligned}$$

Clearly, $$G\left( -x\right) =G\left( x\right) ,$$ and

4 $$\begin{aligned} G\left( 0\right) =\frac{{\textrm{e}}^{\mu }-1}{2\left( {\textrm{e}}^{\mu }+1\right) }. \end{aligned}$$

In [20], we prove that

$G^{{\prime }}\left( x\right) <0$ for $x>0,$ so that

$G\left( x\right)$ is strictly decreasing on $\left( 0,+\infty \right) .$

Clearly, then $G\left( x\right)$ is strictly increasing on $\left( -\infty ,0\right) ,$ along with $G^{{\prime }}\left( 0\right) =0.$

Also, it holds $G\left( \infty \right) =G\left( -\infty \right) =0.$

Conclusion, *G* is a bell symmetric function with maximum as in (4)

$$\begin{aligned} G\left( 0\right) =\frac{{\textrm{e}}^{\mu }-1}{2\left( {\textrm{e}}^{\mu }+1\right) },\quad \mu >0. \end{aligned}$$

We mention

### Theorem 2.1

[20] We have

5 $$\begin{aligned} \sum _{i=-\infty }^{\infty }G\left( x-i\right) =1,\quad \forall ~x\in {\mathbb {R}}. \end{aligned}$$

### Theorem 2.2

[20] It holds

6 $$\begin{aligned} \int _{-\infty }^{\infty }G\left( x\right) {\textrm{d}}x=1, \end{aligned}$$

so that *G* is a density function.

### Theorem 2.3

[20] Let $0<\alpha <1,$ $\mu >0$ and $n\in {\mathbb {N}}$ with $n^{1-\alpha }>2.$ It holds

7 $$\begin{aligned} \sum _{\left\{ \begin{array}{c} k=-\infty \\ :\left| nx-k\right| \ge n^{1-\alpha } \end{array} \right. }^{\infty }G\left( nx-k\right) <\frac{1}{{\textrm{e}}^{\mu \left( n^{1-\alpha }-2\right) }},\quad \mu >0. \end{aligned}$$

Denote by $\left\lfloor \cdot \right\rfloor$ the integral part of the number and by $\left\lceil \cdot \right\rceil$ the ceiling of the number.

### Theorem 2.4

[20] Let $\left[ a,b\right] \subset {\mathbb {R}}$ and $n\in {\mathbb {N}},$ so that $\left\lceil na\right\rceil \le \left\lfloor nb\right\rfloor .$ It holds

8 $$\begin{aligned} \frac{1}{\sum _{k=\left\lceil na\right\rceil }^{\left\lfloor nb\right\rfloor }G\left( nx-k\right) }<\frac{4\left( 1+{\textrm{e}}^{-2\mu }\right) }{ 1-{\textrm{e}}^{-2\mu }},\quad \mu >0 \end{aligned}$$

$$\forall $$ $$x\in \left[ a,b\right] .$$

We make

### Remark 2.5

[20]

(i) We have that
  9 $$\begin{aligned} \underset{n\rightarrow \infty }{\lim }\sum _{k=\left\lceil na\right\rceil }^{\left\lfloor nb\right\rfloor }G\left( nx-k\right) \ne 1, \end{aligned}$$
  for at least some $x\in \left[ a,b\right] .$
(ii) Let $\left[ a,b\right] \subset {\mathbb {R}}.$ For large *n*,  we always have $\left\lceil na\right\rceil \le \left\lfloor nb\right\rfloor .$ Also, $a\le \frac{k}{n}\le b,$ iff $\left\lceil na\right\rceil \le k\le \left\lfloor nb\right\rfloor .$

In general, it holds

10 $$\begin{aligned} \sum _{k=\left\lceil na\right\rceil }^{\left\lfloor nb\right\rfloor }G\left( nx-k\right) \le 1. \end{aligned}$$

We introduce

11 $$\begin{aligned} Z\left( x_{1},\ldots ,x_{N}\right) :=Z\left( x\right) :=\prod _{i=1}^{N}G\left( x_{i}\right) ,\quad x=\left( x_{1},\ldots ,x_{N}\right) \in {\mathbb {R}}^{N},\ N\in {\mathbb {N}}. \end{aligned}$$

It has the properties

(i) $$Z\left( x\right) >0,$$ $$\forall $$ $$x\in {\mathbb {R}}^{N},$$
(ii) 12 $$\begin{aligned} \sum _{k=-\infty }^{\infty }Z\left( x-k\right) :=\sum _{k_{1}=-\infty }^{\infty }\sum _{k_{2}=-\infty }^{\infty }\cdots \sum _{k_{N}=-\infty }^{\infty }Z\left( x_{1}-k_{1},\ldots ,x_{N}-k_{N}\right) =1, \end{aligned}$$
  where $$k:=\left( k_{1},\ldots ,k_{n}\right) \in \mathbb {Z}^{N},$$ $$\forall $$ $$ x\in {\mathbb {R}}^{N};$$
  hence
(iii) 13 $$\begin{aligned} \sum _{k=-\infty }^{\infty }Z\left( nx-k\right) =1 \end{aligned}$$
  $$\forall $$ $$x\in {\mathbb {R}}^{N};$$ $$n\in {\mathbb {N}},$$
  and
(iv) 14 $$\begin{aligned} \int _{{\mathbb {R}}^{N}}Z\left( x\right) {\textrm{d}}x=1, \end{aligned}$$
  that is, *Z* is a multivariate density function.

Here, denote $\left\| x\right\| _{\infty }:=\max \left\{ \left| x_{1}\right| ,\ldots ,\left| x_{N}\right| \right\} ,$ $x\in \mathbb {R }^{N},$ also set $\infty :=\left( \infty ,\ldots ,\infty \right) ,$ $-\infty :=\left( -\infty ,\ldots ,-\infty \right)$ upon the multivariate context, and

15 $$\begin{aligned} \begin{array}{c} \left\lceil na\right\rceil :=\left( \left\lceil na_{1}\right\rceil ,\ldots ,\left\lceil na_{N}\right\rceil \right) , \\ \left\lfloor nb\right\rfloor :=\left( \left\lfloor nb_{1}\right\rfloor ,\ldots ,\left\lfloor nb_{N}\right\rfloor \right) , \end{array} \end{aligned}$$

where $$a:=\left( a_{1},\ldots ,a_{N}\right) ,$$ $$b:=\left( b_{1},\ldots ,b_{N}\right) .$$

We obviously see that

16 $$\begin{aligned}{} & {} \sum _{k=\left\lceil na\right\rceil }^{\left\lfloor nb\right\rfloor }Z\left( nx-k\right) =\sum _{k=\left\lceil na\right\rceil }^{\left\lfloor nb\right\rfloor }\left( \prod _{i=1}^{N}G\left( nx_{i}-k_{i}\right) \right) \nonumber \\{} & {} \quad =\sum _{k_{1}=\left\lceil na_{1}\right\rceil }^{\left\lfloor nb_{1}\right\rfloor }\ldots \sum _{k_{N}=\left\lceil na_{N}\right\rceil }^{\left\lfloor nb_{N}\right\rfloor }\left( \prod _{i=1}^{N}G\left( nx_{i}-k_{i}\right) \right) =\prod _{i=1}^{N}\left( \sum _{k_{i}=\left\lceil na_{i}\right\rceil }^{\left\lfloor nb_{i}\right\rfloor }G\left( nx_{i}-k_{i}\right) \right) . \end{aligned}$$

For $0<\beta <1$ and $n\in {\mathbb {N}},$ a fixed $x\in {\mathbb {R}}^{N},$ we have that

17 $$\begin{aligned}{} & {} \sum _{k=\left\lceil na\right\rceil }^{\left\lfloor nb\right\rfloor }Z\left( nx-k\right) \nonumber \\{} & {} \quad = \sum _{\left\{ \begin{array}{c} k=\left\lceil na\right\rceil \\ \left\| \frac{k}{n}-x\right\| _{\infty }\le \frac{1}{n^{\beta }} \end{array} \right. }^{\left\lfloor nb\right\rfloor }Z\left( nx-k\right) +\sum _{\left\{ \begin{array}{c} k=\left\lceil na\right\rceil \\ \left\| \frac{k}{n}-x\right\| _{\infty }>\frac{1}{n^{\beta }} \end{array} \right. }^{\left\lfloor nb\right\rfloor }Z\left( nx-k\right) . \end{aligned}$$

In the last two sums, the counting is over disjoint vector sets of *k*’s, because the condition $\left\| \frac{k}{n}-x\right\| _{\infty }>\frac{1 }{n^{\beta }}$ implies that there exists at least one $\left| \frac{k_{r} }{n}-x_{r}\right| >\frac{1}{n^{\beta }},$ where $r\in \left\{ 1,\ldots ,N\right\} .$

(v) As in, Theorem 2.3, we derive that

18 $$\begin{aligned} \sum _{\left\{ \begin{array}{c} k=\left\lceil na\right\rceil \\ \left\| \frac{k}{n}-x\right\| _{\infty }>\frac{1}{n^{\beta }} \end{array} \right. }^{\left\lfloor nb\right\rfloor }Z\left( nx-k\right) \overset{(7)}{<}\frac{1}{{\textrm{e}}^{\mu \left( n^{1-\beta }-2\right) }}, \ 0<\beta <1, \mu >0, \end{aligned}$$

with $$n\in {\mathbb {N}}:n^{1-\beta }>2,$$ $$x\in \prod _{i=1}^{N}\left[ a_{i},b_{i}\right] .$$

(vi) By Theorem 2.4, we get that

19 $$\begin{aligned} 0<\frac{1}{\sum _{k=\left\lceil na\right\rceil }^{\left\lfloor nb\right\rfloor }Z\left( nx-k\right) }<\left( \frac{4\left( 1+{\textrm{e}}^{-2\mu }\right) }{1-{\textrm{e}}^{-2\mu }}\right) ^{N}, \end{aligned}$$

$$\mu >0,$$ $$\forall $$ $$x\in \left( \prod _{i=1}^{N}\left[ a_{i},b_{i}\right] \right) ,$$ $$n\in {\mathbb {N}}.$$

It is also clear that

(vii)

20 $$\begin{aligned} \sum _{\left\{ \begin{array}{c} k=-\infty \\ \left\| \frac{k}{n}-x\right\| _{\infty }>\frac{1}{n^{\beta }} \end{array} \right. }^{\infty }Z\left( nx-k\right) <\frac{1}{{\textrm{e}}^{\mu \left( n^{1-\beta } ~ -2\right) }}, \end{aligned}$$

$$\mu >0,0<\beta <1,$$ $$n\in {\mathbb {N}}:n^{1-\beta }>2,$$ $$x\in {\mathbb {R}}^{N}.$$

Furthermore, it holds

21 $$\begin{aligned} \underset{n\rightarrow \infty }{\lim }\sum _{k=\left\lceil na\right\rceil }^{\left\lfloor nb\right\rfloor }Z\left( nx-k\right) \ne 1, \end{aligned}$$

for at least some $x\in \left( \prod _{i=1}^{N}\left[ a_{i},b_{i}\right] \right) .$

Here, $\left( X,\left\| \cdot \right\| _{\gamma }\right)$ is a Banach space.

Let $f\in C\left( \prod _{i=1}^{N}\left[ a_{i},b_{i}\right] ,X\right) ,$ $x=\left( x_{1},\ldots ,x_{N}\right) \in \prod _{i=1}^{N}\left[ a_{i},b_{i}\right] ,$ $n\in {\mathbb {N}},$ such that $\left\lceil na_{i}\right\rceil \le \left\lfloor nb_{i}\right\rfloor ,$ $i=1,\ldots ,N.$

We introduce and define the following multivariate linear normalized neural network operator $(x:=\left( x_{1},\ldots ,x_{N}\right) \in \left( \prod _{i=1}^{N}\left[ a_{i},b_{i}\right] \right) )$:

22 $$\begin{aligned}{} & {} L_{n}\left( f,x_{1},\ldots ,x_{N}\right) :=L_{n}\left( f,x\right) :=\frac{ \sum _{k=\left\lceil na\right\rceil }^{\left\lfloor nb\right\rfloor }f\left( \frac{k}{n}\right) Z\left( nx-k\right) }{\sum _{k=\left\lceil na\right\rceil }^{\left\lfloor nb\right\rfloor }Z\left( nx-k\right) }\nonumber \\{} & {} \quad = \frac{\sum _{k_{1}=\left\lceil na_{1}\right\rceil }^{\left\lfloor nb_{1}\right\rfloor }\sum _{k_{2}=\left\lceil na_{2}\right\rceil }^{\left\lfloor nb_{2}\right\rfloor }\cdots \sum _{k_{N}=\left\lceil na_{N}\right\rceil }^{\left\lfloor nb_{N}\right\rfloor }f\left( \frac{k_{1}}{ n},\ldots ,\frac{k_{N}}{n}\right) \left( \prod _{i=1}^{N}G\left( nx_{i}-k_{i}\right) \right) }{\prod _{i=1}^{N}\left( \sum _{k_{i}=\left\lceil na_{i}\right\rceil }^{\left\lfloor nb_{i}\right\rfloor }G\left( nx_{i}-k_{i}\right) \right) }. \end{aligned}$$

For large enough $n\in {\mathbb {N}},$ we always obtain $\left\lceil na_{i}\right\rceil \le \left\lfloor nb_{i}\right\rfloor ,$ $i=1,\ldots ,N.$ Also, $a_{i}\le \frac{k_{i}}{n}\le b_{i},$ iff $\left\lceil na_{i}\right\rceil \le k_{i}\le \left\lfloor nb_{i}\right\rfloor ,$ $i=1,\ldots ,N.$

When $g\in C\left( \prod _{i=1}^{N}\left[ a_{i},b_{i}\right] \right) ,$ we define the companion operator

23 $$\begin{aligned} {\widetilde{L}}_{n}\left( g,x\right) :=\frac{\sum _{k=\left\lceil na\right\rceil }^{\left\lfloor nb\right\rfloor }g\left( \frac{k}{n}\right) Z\left( nx-k\right) }{\sum _{k=\left\lceil na\right\rceil }^{\left\lfloor nb\right\rfloor }Z\left( nx-k\right) }. \end{aligned}$$

Clearly, ${\widetilde{L}}_{n}$ is a positive linear operator. We have that

$$\begin{aligned} {\widetilde{L}}_{n}\left( 1,x\right) =1, \quad \forall ~x\in \left( \prod _{i=1}^{N}\left[ a_{i},b_{i}\right] \right) . \end{aligned}$$

Notice that $L_{n}\left( f\right) \in C\left( \prod _{i=1}^{N}\left[ a_{i},b_{i}\right] ,X\right)$ and ${\widetilde{L}}_{n}\left( g\right) \in C\left( \prod _{i=1}^{N}\left[ a_{i},b_{i}\right] \right) .$

Furthermore, it holds

24 $$\begin{aligned} \left\| L_{n}\left( f,x\right) \right\| _{\gamma }\le \frac{ \sum _{k=\left\lceil na\right\rceil }^{\left\lfloor nb\right\rfloor }\left\| f\left( \frac{k}{n}\right) \right\| _{\gamma }Z\left( nx-k\right) }{\sum _{k=\left\lceil na\right\rceil }^{\left\lfloor nb\right\rfloor }Z\left( nx-k\right) }={\widetilde{L}}_{n}\left( \left\| f\right\| _{\gamma },x\right) \end{aligned}$$

$$\forall $$ $$x\in \prod _{i=1}^{N}\left[ a_{i},b_{i}\right] .$$

Clearly, $$\left\| f\right\| _{\gamma }\in C\left( \prod _{i=1}^{N} \left[ a_{i},b_{i}\right] \right) .$$

Therefore, we have that

25 $$\begin{aligned} \left\| L_{n}\left( f,x\right) \right\| _{\gamma }\le {\widetilde{L}} _{n}\left( \left\| f\right\| _{\gamma },x\right) , \end{aligned}$$

$$\forall $$ $$x\in \prod _{i=1}^{N}\left[ a_{i},b_{i}\right] ,$$ $$\forall $$ $$ n\in {\mathbb {N}},$$ $$\forall $$ $$f\in C\left( \prod _{i=1}^{N}\left[ a_{i},b_{i} \right] ,X\right) .$$

Let $c\in X$ and $g\in C\left( \prod _{i=1}^{N}\left[ a_{i},b_{i}\right] \right) ,$ then $cg\in C\left( \prod _{i=1}^{N}\left[ a_{i},b_{i}\right] ,X\right) .$

Furthermore, it holds

26 $$\begin{aligned} L_{n}\left( cg,x\right) =c{\widetilde{L}}_{n}\left( g,x\right) , \quad \forall ~x\in \prod _{i=1}^{N}\left[ a_{i},b_{i}\right] . \end{aligned}$$

Since ${\widetilde{L}}_{n}\left( 1\right) =1,$ we get that

27 $$\begin{aligned} L_{n}\left( c\right) =c,\quad \forall ~c\in X. \end{aligned}$$

We call ${\widetilde{L}}_{n}$ the companion operator of $L_{n}.$

For convenience, we call

28 $$\begin{aligned}{} & {} L_{n}^{*}\left( f,x\right) :=\sum _{k=\left\lceil na\right\rceil }^{\left\lfloor nb\right\rfloor }f\left( \frac{k}{n}\right) Z\left( nx-k\right) \nonumber \\{} & {} \quad =\sum _{k_{1}=\left\lceil na_{1}\right\rceil }^{\left\lfloor nb_{1}\right\rfloor }\sum _{k_{2}=\left\lceil na_{2}\right\rceil }^{\left\lfloor nb_{2}\right\rfloor }\cdots \sum _{k_{N}=\left\lceil na_{N}\right\rceil }^{\left\lfloor nb_{N}\right\rfloor }f\left( \frac{k_{1}}{ n},\ldots ,\frac{k_{N}}{n}\right) \left( \prod _{i=1}^{N}G\left( nx_{i}-k_{i}\right) \right) \end{aligned}$$

$$\forall $$ $$x\in \left( \prod _{i=1}^{N}\left[ a_{i},b_{i}\right] \right) .$$

That is

29 $$\begin{aligned} L_{n}\left( f,x\right) :=\frac{L_{n}^{*}\left( f,x\right) }{ \sum _{k=\left\lceil na\right\rceil }^{\left\lfloor nb\right\rfloor }Z\left( nx-k\right) } \end{aligned}$$

$$\forall $$ $$x\in \left( \prod _{i=1}^{N}\left[ a_{i},b_{i}\right] \right) ,$$ $$ n\in {\mathbb {N}}.$$

Hence

30 $$\begin{aligned} L_{n}\left( f,x\right) -f\left( x\right) =\frac{L_{n}^{*}\left( f,x\right) -f\left( x\right) \left( \sum _{k=\left\lceil na\right\rceil }^{\left\lfloor nb\right\rfloor }Z\left( nx-k\right) \right) }{ \sum _{k=\left\lceil na\right\rceil }^{\left\lfloor nb\right\rfloor }Z\left( nx-k\right) }. \end{aligned}$$

Consequently, we derive

31 $$\begin{aligned} \left\| L_{n}\left( f,x\right) -f\left( x\right) \right\| _{\gamma } \overset{(19)}{\le }\left( \frac{4\left( 1+{\textrm{e}}^{-2\mu }\right) }{ 1-{\textrm{e}}^{-2\mu }}\right) ^{N}\left\| L_{n}^{*}\left( f,x\right) -f\left( x\right) \sum _{k=\left\lceil na\right\rceil }^{\left\lfloor nb\right\rfloor }Z\left( nx-k\right) \right\| _{\gamma } \end{aligned}$$

$$\forall $$ $$x\in \left( \prod _{i=1}^{N}\left[ a_{i},b_{i}\right] \right) .$$

We will estimate the right-hand side of (31).

For the last and others, we need the following.

### Definition 2.6

[15, p. 274] Let *M* be a convex and compact subset of $\left( {\mathbb {R}}^{N},\left\| \cdot \right\| _{p}\right) ,$ $p\in \left[ 1,\infty \right] ,$ and $\left( X,\left\| \cdot \right\| _{\gamma }\right)$ be a Banach space. Let $f\in C\left( M,X\right) .$ We define the first modulus of continuity of *f* as

32 $$\begin{aligned} \omega _{1}\left( f,\delta \right) :=\underset{ \begin{array}{c} x,y\in M: \\ \left\| x-y\right\| _{p}\le \delta \end{array} }{\sup }\left\| f\left( x\right) -f\left( y\right) \right\| _{\gamma } , \ 0<\delta \le \text {diam}\left( M\right) . \end{aligned}$$

If $$\delta >\hbox {diam}\left( M\right) ,$$ then

33 $$\begin{aligned} \omega _{1}\left( f,\delta \right) =\omega _{1}\left( f,\text {diam}\left( M\right) \right) . \end{aligned}$$

Notice $\omega _{1}\left( f,\delta \right)$ is increasing in $\delta >0.$ For $f\in C_{B}\left( M,X\right)$ (continuous and bounded functions), $\omega _{1}\left( f,\delta \right)$ is defined similarly.

### Lemma 2.7

[15, p. 274] We have $\omega _{1}\left( f,\delta \right) \rightarrow 0$ as $\delta \downarrow 0,$ iff $f\in C\left( M,X\right) ,$ where *M* is a convex compact subset of $\left( {\mathbb {R}}^{N},\left\| \cdot \right\| _{p}\right) ,$ $p\in \left[ 1,\infty \right] .$

Clearly, we have also: $f\in C_{U}\left( {\mathbb {R}}^{N},X\right)$ (uniformly continuous functions), iff $\omega _{1}\left( f,\delta \right) \rightarrow 0$ as $\delta \downarrow 0,$ where $\omega _{1}$ is defined similarly to (32). The space $C_{B}\left( {\mathbb {R}}^{N},X\right)$ denotes the continuous and bounded functions on ${\mathbb {R}}^{N}.$

When $f\in C_{B}\left( {\mathbb {R}}^{N},X\right) ,$ we define

34 $$\begin{aligned}{} & {} B_{n}\left( f,x\right) :=B_{n}\left( f,x_{1},\ldots ,x_{N}\right) :=\sum _{k=-\infty }^{\infty }f\left( \frac{k}{n}\right) Z\left( nx-k\right) \nonumber \\{} & {} \quad := \sum _{k_{1}=-\infty }^{\infty }\sum _{k_{2}=-\infty }^{\infty }\cdots \sum _{k_{N}=-\infty }^{\infty }f\left( \frac{k_{1}}{n},\frac{k_{2}}{n} ,\ldots ,\frac{k_{N}}{n}\right) \left( \prod _{i=1}^{N}G\left( nx_{i}-k_{i}\right) \right) , \end{aligned}$$

$n\in {\mathbb {N}},$ $\forall$ $x\in {\mathbb {R}}^{N},$ $N\in {\mathbb {N}},$ the multivariate quasi-interpolation neural network operator.

Also, for $f\in C_{B}\left( {\mathbb {R}}^{N},X\right) ,$ we define the multivariate Kantorovich-type neural network operator

35 $$\begin{aligned}{} & {} C_{n}\left( f,x\right) :=C_{n}\left( f,x_{1},\ldots ,x_{N}\right) :=\sum _{k=-\infty }^{\infty }\left( n^{N}\int _{\frac{k}{n}}^{\frac{k+1}{n} }f\left( t\right) {\textrm{d}}t\right) Z\left( nx-k\right) \nonumber \\{} & {} \quad = \sum _{k_{1}=-\infty }^{\infty }\sum _{k_{2}=-\infty }^{\infty }\cdots \sum _{k_{N}=-\infty }^{\infty }\left( n^{N}\int _{\frac{k_{1}}{n}}^{\frac{ k_{1}+1}{n}}\int _{\frac{k_{2}}{n}}^{\frac{k_{2}+1}{n}}\cdots \int _{\frac{k_{N}}{n }}^{\frac{k_{N}+1}{n}}f\left( t_{1},\ldots ,t_{N}\right) {\textrm{d}}t_{1}\ldots {\textrm{d}}t_{N}\right) \nonumber \\{} & {} \qquad \cdot \left( \prod _{i=1}^{N}G\left( nx_{i}-k_{i}\right) \right) , \end{aligned}$$

$$n\in {\mathbb {N}},\ \forall $$ $$x\in {\mathbb {R}}^{N}.$$

Again, for $f\in C_{B}\left( {\mathbb {R}}^{N},X\right) ,$ $N\in {\mathbb {N}},$ we define the multivariate neural network operator of quadrature-type $D_{n}\left( f,x\right) ,$ $n\in {\mathbb {N}},$ as follows.

Let $\theta =\left( \theta _{1},\ldots ,\theta _{N}\right) \in {\mathbb {N}}^{N},$ $r=\left( r_{1},\ldots ,r_{N}\right) \in \mathbb {Z}_{+}^{N},$ $w_{r}=w_{r_{1},r_{2},\ldots r_{N}}\ge 0,$ such that $\sum _{r=0}^{\theta }w_{r}=\sum _{r_{1}=0}^{\theta _{1}}\sum _{r_{2}=0}^{\theta _{2}}\ldots \sum _{r_{N}=0}^{\theta _{N}}w_{r_{1},r_{2},\ldots r_{N}}=1;$ $k\in \mathbb {Z}^{N}$ and

36 $$\begin{aligned}{} & {} \delta _{nk}\left( f\right) :=\delta _{n,k_{1},k_{2},\ldots ,k_{N}}\left( f\right) :=\sum _{r=0}^{\theta }w_{r}f\left( \frac{k}{n}+\frac{r}{ n\theta }\right) \nonumber \\{} & {} \quad = \sum _{r_{1}=0}^{\theta _{1}}\sum _{r_{2}=0}^{\theta _{2}}\cdots \sum _{r_{N}=0}^{\theta _{N}}w_{r_{1},r_{2},\ldots r_{N}}f\left( \frac{k_{1}}{n}+\frac{r_{1}}{n\theta _{1}},\frac{k_{2}}{n}+\frac{r_{2}}{ n\theta _{2}},\ldots ,\frac{k_{N}}{n}+\frac{r_{N}}{n\theta _{N}}\right) , \end{aligned}$$

where $$\frac{r}{\theta }:=\left( \frac{r_{1}}{\theta _{1}},\frac{r_{2}}{ \theta _{2}},\ldots ,\frac{r_{N}}{\theta _{N}}\right) .$$

We set

37 $$\begin{aligned}{} & {} D_{n}\left( f,x\right) :=D_{n}\left( f,x_{1},\ldots ,x_{N}\right) :=\sum _{k=-\infty }^{\infty }\delta _{nk}\left( f\right) Z\left( nx-k\right) \nonumber \\{} & {} \quad = \sum _{k_{1}=-\infty }^{\infty }\sum _{k_{2}=-\infty }^{\infty }\cdots \sum _{k_{N}=-\infty }^{\infty }\delta _{n,k_{1},k_{2},\ldots ,k_{N}}\left( f\right) \left( \prod _{i=1}^{N}G\left( nx_{i}-k_{i}\right) \right) \end{aligned}$$

$$\forall $$ $$x\in {\mathbb {R}}^{N}.$$

In this article, we study the approximation properties of $L_{n},B_{n},C_{n},$ $D_{n}$ neural network operators and as well of their iterates. That is, the quantitative pointwise and uniform convergence of these operators to the unit operator *I*.

## Multivariate Richards’s curve neural network approximations

Here, we present several vectorial neural network approximations to Banach space valued functions given with rates.

We give

### Theorem 3.1

Let $f\in C\left( \prod _{i=1}^{N}\left[ a_{i},b_{i}\right] ,X\right) ,$ $0<\beta <1,$ $\mu >0,$ $x\in \left( \prod _{i=1}^{N}\left[ a_{i},b_{i}\right] \right) ,N,n\in {\mathbb {N}}$ with $n^{1-\beta }>2.$ Then

1. 38 $$\begin{aligned} \left\| L_{n}\left( f,x\right) -f\left( x\right) \right\| _{\gamma }\le \left( \frac{4\left( 1+{\textrm{e}}^{-2\mu }\right) }{1-{\textrm{e}}^{-2\mu }}\right) ^{N} \left[ \omega _{1}\left( f,\frac{1}{n^{\beta }}\right) +\frac{2\left\| \left\| f\right\| _{\gamma }\right\| _{\infty }}{{\textrm{e}}^{\mu \left( n^{1-\beta }-2\right) }}\right] =:\lambda _{1}\left( n\right) , \end{aligned}$$
  and
2. 39 $$\begin{aligned} \left\| \left\| L_{n}\left( f\right) -f\right\| _{\gamma }\right\| _{\infty }\le \lambda _{1}\left( n\right) . \end{aligned}$$
  We notice that $\lim _{n\rightarrow \infty }L_{n}\left( f\right) \overset{\left\| \cdot \right\| _{\gamma }}{=}f,$ pointwise and uniformly.

Above $\omega _{1}$ is with respect to $p=\infty$ and the speed of convergence is $\max \left( \frac{1}{n^{\beta }},\frac{2}{{\textrm{e}}^{\mu \left( n^{1-\beta }-2\right) }}\right) =\frac{1}{n^{\beta }}.$

### Proof

We observe that

40 $$\begin{aligned}{} & {} \Delta \left( x\right) :=L_{n}^{*}\left( f,x\right) -f\left( x\right) \sum _{k=\left\lceil na\right\rceil }^{\left\lfloor nb\right\rfloor }Z\left( nx-k\right) \nonumber \\{} & {} \quad = \sum _{k=\left\lceil na\right\rceil }^{\left\lfloor nb\right\rfloor }f\left( \frac{k}{n}\right) Z\left( nx-k\right) -\sum _{k=\left\lceil na\right\rceil }^{\left\lfloor nb\right\rfloor }f\left( x\right) Z\left( nx-k\right) \nonumber \\{} & {} \quad = \sum _{k=\left\lceil na\right\rceil }^{\left\lfloor nb\right\rfloor }\left( f\left( \frac{k}{n}\right) -f\left( x\right) \right) Z\left( nx-k\right) . \end{aligned}$$

Thus

41 $$\begin{aligned}{} & {} \left\| \Delta \left( x\right) \right\| _{\gamma }\le \sum _{k=\left\lceil na\right\rceil }^{\left\lfloor nb\right\rfloor }\left\| f\left( \frac{k}{n}\right) -f\left( x\right) \right\| _{\gamma }Z\left( nx-k\right) \nonumber \\{} & {} \quad = \sum _{\left\{ \begin{array}{c} k=\left\lceil na\right\rceil \\ \left\| \frac{k}{n}-x\right\| _{\infty }\le \frac{1}{n^{\beta }} \end{array} \right. }^{\left\lfloor nb\right\rfloor }\left\| f\left( \frac{k}{n} \right) -f\left( x\right) \right\| _{\gamma }Z\left( nx-k\right) \nonumber \\{} & {} \qquad + \sum _{\left\{ \begin{array}{c} k=\left\lceil na\right\rceil \\ \left\| \frac{k}{n}-x\right\| _{\infty }>\frac{1}{n^{\beta }} \end{array} \right. }^{\left\lfloor nb\right\rfloor }\left\| f\left( \frac{k}{n} \right) -f\left( x\right) \right\| _{\gamma }Z\left( nx-k\right) \nonumber \\{} & {} \quad \overset{(13)}{\le } \omega _{1}\left( f,\frac{1}{n^{\beta }}\right) +2\left\| \left\| f\right\| _{\gamma }\right\| _{\infty }\sum _{\left\{ \begin{array}{c} k=\left\lceil na\right\rceil \\ \left\| \frac{k}{n}-x\right\| _{\infty }>\frac{1}{n^{\beta }} \end{array} \right. }^{\left\lfloor nb\right\rfloor }Z\left( nx-k\right) \nonumber \\{} & {} \quad \overset{(18)}{\le } \omega _{1}\left( f,\frac{1}{n^{\beta }}\right) +\frac{2\left\| \left\| f\right\| _{\gamma }\right\| _{\infty }}{{\textrm{e}}^{\mu \left( n^{1-\beta }-2\right) }},\quad 0<\beta <1, \mu >0. \end{aligned}$$

So that

42 $$\begin{aligned} \left\| \Delta \left( x\right) \right\| _{\gamma }\le \omega _{1}\left( f,\frac{1}{n^{\beta }}\right) +\frac{2\left\| \left\| f\right\| _{\gamma }\right\| _{\infty }}{{\textrm{e}}^{\mu \left( n^{1-\beta }-2\right) }}. \end{aligned}$$

Now, using (31), we finish the proof. $\square$

We make

### Remark 3.2

[15, pp. 263–266] Let $\left( {\mathbb {R}}^{N},\left\| \cdot \right\| _{p}\right) ,$ $N\in {\mathbb {N}}$; where $\left\| \cdot \right\| _{p}$ is the $L_{p}$-norm, $1\le p\le \infty .$ ${\mathbb {R}} ^{N}$ is a Banach space, and $\left( {\mathbb {R}}^{N}\right) ^{j}$ denotes the *j*-fold product space ${\mathbb {R}}^{N}\times \cdots \times {\mathbb {R}}^{N}$ endowed with the max-norm $\left\| x\right\| _{\left( {\mathbb {R}} ^{N}\right) ^{j}}:=\max _{1\le \lambda \le j}\left\| x_{\lambda }\right\| _{p},$ where $x:=\left( x_{1},\ldots ,x_{j}\right) \in \left( {\mathbb {R}}^{N}\right) ^{j}.$

Let $\left( X,\left\| \cdot \right\| _{\gamma }\right)$ be a general Banach space. Then, the space $V_{j}:=V_{j}\left( \left( {\mathbb {R}} ^{N}\right) ^{j};X\right)$ of all *j*-multilinear continuous maps $g:\left( {\mathbb {R}}^{N}\right) ^{j}\rightarrow X,$ $j=1,\ldots ,m,$ is a Banach space with norm

43 $$\begin{aligned} \left\| g\right\| :=\left\| g\right\| _{V_{j}}:=\underset{\left( \left\| x\right\| _{\left( {\mathbb {R}}^{N}\right) ^{j}}=1\right) }{\sup }\left\| g\left( x\right) \right\| _{\gamma }=\sup \frac{\left\| g\left( x\right) \right\| _{\gamma }}{\left\| x_{1}\right\| _{p}\cdots \left\| x_{j}\right\| _{p}}. \end{aligned}$$

Let *M* be a non-empty convex and compact subset of ${\mathbb {R}}^{N}$ and $x_{0}\in M$ is fixed.

Let *O* be an open subset of ${\mathbb {R}}^{N}:M\subset O.$ Let $f:O\rightarrow X$ be a continuous function, whose Fréchet derivatives (see [29]) $f^{\left( j\right) }:O\rightarrow V_{j}=V_{j}\left( \left( {\mathbb {R}}^{N}\right) ^{j};X\right)$ exist and are continuous for $1\le j\le m,$ $m\in {\mathbb {N}}.$

Call $$\left( x-x_{0}\right) ^{j}:=\left( x-x_{0},\ldots ,x-x_{0}\right) \in \left( {\mathbb {R}}^{N}\right) ^{j},$$ $$x\in M.$$

We will work with $f|_{M}.$

Then, by Taylor’s formula [21, 29, p. 124], we get

44 $$\begin{aligned} f\left( x\right) =\sum _{j=0}^{m}\frac{f^{\left( j\right) }\left( x_{0}\right) \left( x-x_{0}\right) ^{j}}{j!}+R_{m}\left( x,x_{0}\right) ,\quad \text {all }x\in M, \end{aligned}$$

where the remainder is the Riemann integral

45 $$\begin{aligned} R_{m}\left( x,x_{0}\right) :=\int _{0}^{1}\frac{\left( 1-u\right) ^{m-1}}{ \left( m-1\right) !}\left( f^{\left( m\right) }\left( x_{0}+u\left( x-x_{0}\right) \right) -f^{\left( m\right) }\left( x_{0}\right) \right) \left( x-x_{0}\right) ^{m}{\textrm{d}}u; \end{aligned}$$

here, we set $f^{\left( 0\right) }\left( x_{0}\right) \left( x-x_{0}\right) ^{0}=f\left( x_{0}\right) .$

We consider

46 $$\begin{aligned} w:=\omega _{1}\left( f^{\left( m\right) },h\right) :=\underset{\left\| x-y\right\| _{p}\le h}{\underset{x,y\in M:}{\sup }}\left\| f^{\left( m\right) }\left( x\right) -f^{\left( m\right) }\left( y\right) \right\| , \end{aligned}$$

$$h>0.$$

We obtain

47 $$\begin{aligned}{} & {} \left\| \left( f^{\left( m\right) }\left( x_{0}+u\left( x-x_{0}\right) \right) -f^{\left( m\right) }\left( x_{0}\right) \right) \left( x-x_{0}\right) ^{m}\right\| _{\gamma }\nonumber \\{} & {} \quad \le \left\| f^{\left( m\right) }\left( x_{0}+u\left( x-x_{0}\right) \right) -f^{\left( m\right) }\left( x_{0}\right) \right\| \cdot \left\| x-x_{0}\right\| _{p}^{m}\nonumber \\{} & {} \quad \le w\left\| x-x_{0}\right\| _{p}^{m}\left\lceil \frac{u\left\| x-x_{0}\right\| _{p}}{h}\right\rceil , \end{aligned}$$

by Lemma 7.1.1, [1, p. 208], where $\left\lceil \cdot \right\rceil$ is the ceiling.

Therefore, for all $x\in M$ (see [1, pp. 121–122])

48 $$\begin{aligned}{} & {} \left\| R_{m}\left( x,x_{0}\right) \right\| _{\gamma }\le w\left\| x-x_{0}\right\| _{p}^{m}\int _{0}^{1}\left\lceil \frac{u\left\| x-x_{0}\right\| _{p}}{h}\right\rceil \frac{\left( 1-u\right) ^{m-1}}{ \left( m-1\right) !}{\textrm{d}}u\nonumber \\{} & {} \quad =w\Phi _{m}\left( \left\| x-x_{0}\right\| _{p}\right) \end{aligned}$$

by a change of variable, where

49 $$\begin{aligned} \Phi _{m}\left( t\right) :=\int _{0}^{\left| t\right| }\left\lceil \frac{s}{h}\right\rceil \frac{\left( \left| t\right| -s\right) ^{m-1} }{\left( m-1\right) !}{\textrm{d}}s=\frac{1}{m!}\left( \sum _{j=0}^{\infty }\left( \left| t\right| -jh\right) _{+}^{m}\right) ,\quad \forall ~ t\in {\mathbb {R}} \end{aligned}$$

is a (polynomial) spline function; see [1, p. 210–211].

Also, from there, we get

50 $$\begin{aligned} \Phi _{m}\left( t\right) \le \left( \frac{\left| t\right| ^{m+1}}{ \left( m+1\right) !h}+\frac{\left| t\right| ^{m}}{2m!}+\frac{ h\left| t\right| ^{m-1}}{8\left( m-1\right) !}\right) ,\quad \forall ~t\in {\mathbb {R}}, \end{aligned}$$

with equality true only at $t=0.$

Therefore, it holds

51 $$\begin{aligned} \left\| R_{m}\left( x,x_{0}\right) \right\| _{\gamma }\le w\left( \frac{\left\| x-x_{0}\right\| _{p}^{m+1}}{\left( m+1\right) !h}+\frac{ \left\| x-x_{0}\right\| _{p}^{m}}{2m!}+\frac{h\left\| x-x_{0}\right\| _{p}^{m-1}}{8\left( m-1\right) !}\right) , \quad \forall ~x\in M. \end{aligned}$$

We have found that

52 $$\begin{aligned}{} & {} \left\| f\left( x\right) -\sum _{j=0}^{m}\frac{f^{\left( j\right) }\left( x_{0}\right) \left( x-x_{0}\right) ^{j}}{j!}\right\| _{\gamma }\nonumber \\{} & {} \quad \le \omega _{1}\left( f^{\left( m\right) },h\right) \left( \frac{\left\| x-x_{0}\right\| _{p}^{m+1}}{\left( m+1\right) !h}+\frac{\left\| x-x_{0}\right\| _{p}^{m}}{2m!}+\frac{h\left\| x-x_{0}\right\| _{p}^{m-1}}{8\left( m-1\right) !}\right) <\infty , \end{aligned}$$

$$\forall $$ $$x,x_{0}\in M.$$

Here, $0<\omega _{1}\left( f^{\left( m\right) },h\right) <\infty ,$ by *M* being compact and $f^{\left( m\right) }$ being continuous on *M*.

One can rewrite (52) as follows:

53 $$\begin{aligned}{} & {} \left\| f\left( \cdot \right) -\sum _{j=0}^{m}\frac{f^{\left( j\right) }\left( x_{0}\right) \left( \cdot -x_{0}\right) ^{j}}{j!}\right\| _{\gamma }\nonumber \\{} & {} \quad \le \omega _{1}\left( f^{\left( m\right) },h\right) \left( \frac{\left\| \cdot -x_{0}\right\| _{p}^{m+1}}{\left( m+1\right) !h}+\frac{\left\| \cdot -x_{0}\right\| _{p}^{m}}{2m!}+\frac{h\left\| \cdot -x_{0}\right\| _{p}^{m-1}}{8\left( m-1\right) !}\right) ,\quad \forall ~x_{0}\in M, \end{aligned}$$

a pointwise functional inequality on *M*.

Here, $\left( \cdot -x_{0}\right) ^{j}$ maps *M* into $\left( {\mathbb {R}} ^{N}\right) ^{j}$ and it is continuous, and also, $f^{\left( j\right) }\left( x_{0}\right)$ maps $\left( {\mathbb {R}}^{N}\right) ^{j}$ into *X* and it is continuous. Hence, their composition $f^{\left( j\right) }\left( x_{0}\right) \left( \cdot -x_{0}\right) ^{j}$ is continuous from *M* into *X*.

Clearly, $f\left( \cdot \right) -\sum _{j=0}^{m}\frac{f^{\left( j\right) }\left( x_{0}\right) \left( \cdot -x_{0}\right) ^{j}}{j!}\in C\left( M,X\right) ,$ and hence, $\left\| f\left( \cdot \right) -\sum _{j=0}^{m}\frac{ f^{\left( j\right) }\left( x_{0}\right) \left( \cdot -x_{0}\right) ^{j}}{j!} \right\| _{\gamma }\in C\left( M\right) .$

Let $\left\{ {\widetilde{S}}_{N}\right\} _{N\in {\mathbb {N}}}$ be a sequence of positive linear operators’ mapping $C\left( M\right)$ into $C\left( M\right) .$

Therefore, we obtain

54 $$\begin{aligned}{} & {} \left( {\widetilde{S}}_{N}\left( \left\| f\left( \cdot \right) -\sum _{j=0}^{m}\frac{f^{\left( j\right) }\left( x_{0}\right) \left( \cdot -x_{0}\right) ^{j}}{j!}\right\| _{\gamma }\right) \right) \left( x_{0}\right) \nonumber \\{} & {} \quad \le \omega _{1}\left( f^{\left( m\right) },h\right) \left[ \frac{\left( {\widetilde{S}}_{N}\left( \left\| \cdot -x_{0}\right\| _{p}^{m+1}\right) \right) \left( x_{0}\right) }{\left( m+1\right) !h}+\frac{\left( \widetilde{S }_{N}\left( \left\| \cdot -x_{0}\right\| _{p}^{m}\right) \right) \left( x_{0}\right) }{2m!}\right. \nonumber \\{} & {} \qquad \left. +\frac{h\left( {\widetilde{S}}_{N}\left( \left\| \cdot -x_{0}\right\| _{p}^{m-1}\right) \right) \left( x_{0}\right) }{8\left( m-1\right) !}\right] \end{aligned}$$

$$\forall $$ $$N\in {\mathbb {N}},$$ $$\forall $$ $$x_{0}\in M.$$

Clearly, (54) is valid when $M=\prod _{i=1}^{N}\left[ a_{i},b_{i} \right]$ and ${\widetilde{S}}_{n}={\widetilde{L}}_{n},$ see (23).

All the above is preparation for the following theorem, where we assume Fr échet differentiability of functions.

This will be a direct application of Theorem 10.2, [15, pp. 268–270]. The operators $L_{n},$ ${\widetilde{L}}_{n}$ fulfill its assumptions; see (22), (23), (25), (26), and (27).

We present the following high-order approximation results.

### Theorem 3.3

Let *O* open subset of $\left( {\mathbb {R}}^{N},\left\| \cdot \right\| _{p}\right) ,$ $p\in \left[ 1,\infty \right] ,$ such that $\prod _{i=1}^{N}\left[ a_{i},b_{i}\right] \subset O\subseteq {\mathbb {R}} ^{N},$ and let $\left( X,\left\| \cdot \right\| _{\gamma }\right)$ be a general Banach space. Let $m\in {\mathbb {N}}$ and $f\in C^{m}\left( O,X\right) ,$ the space of *m*-times continuously Fréchet differentiable functions from *O* into *X*. We study the approximation of $f|_{\prod _{i=1}^{N}\left[ a_{i},b_{i}\right] }.$ Let $x_{0}\in \left( \prod _{i=1}^{N}\left[ a_{i},b_{i}\right] \right)$ and $r>0.$ Then

1. 55 $$\begin{aligned}{} & {} \left\| \left( L_{n}\left( f\right) \right) \left( x_{0}\right) -\sum _{j=0}^{m}\frac{1}{j!}\left( L_{n}\left( f^{\left( j\right) }\left( x_{0}\right) \left( \cdot -x_{0}\right) ^{j}\right) \right) \left( x_{0}\right) \right\| _{\gamma }\nonumber \\{} & {} \quad \le \frac{\omega _{1}\left( f^{\left( m\right) },r\left( \left( {\widetilde{L}} _{n}\left( \left\| \cdot -x_{0}\right\| _{p}^{m+1}\right) \right) \left( x_{0}\right) \right) ^{\frac{1}{m+1}}\right) }{rm!}\left( \left( {\widetilde{L}}_{n}\left( \left\| \cdot -x_{0}\right\| _{p}^{m+1}\right) \right) \left( x_{0}\right) \right) ^{\left( \frac{m}{m+1}\right) }\nonumber \\{} & {} \qquad \times \left[ \frac{1}{\left( m+1\right) }+\frac{r}{2}+\frac{mr^{2}}{8}\right] ; \end{aligned}$$
2. in addition,  if $f^{\left( j\right) }\left( x_{0}\right) =0,$ $j=1,\ldots ,m,$ we have
  56 $$\begin{aligned}{} & {} \left\| \left( L_{n}\left( f\right) \right) \left( x_{0}\right) -f\left( x_{0}\right) \right\| _{\gamma }\nonumber \\{} & {} \quad \le \frac{\omega _{1}\left( f^{\left( m\right) },r\left( \left( {\widetilde{L}} _{n}\left( \left\| \cdot -x_{0}\right\| _{p}^{m+1}\right) \right) \left( x_{0}\right) \right) ^{\frac{1}{m+1}}\right) }{rm!}\left( \left( {\widetilde{L}}_{n}\left( \left\| \cdot -x_{0}\right\| _{p}^{m+1}\right) \right) \left( x_{0}\right) \right) ^{\left( \frac{m}{m+1}\right) }\nonumber \\{} & {} \qquad \times \left[ \frac{1}{\left( m+1\right) }+\frac{r}{2}+\frac{mr^{2}}{8}\right] ; \end{aligned}$$
3. 57 $$\begin{aligned}{} & {} \left\| \left( L_{n}\left( f\right) \right) \left( x_{0}\right) -f\left( x_{0}\right) \right\| _{\gamma }\le \sum _{j=1}^{m}\frac{1}{j!}\left\| \left( L_{n}\left( f^{\left( j\right) }\left( x_{0}\right) \left( \cdot -x_{0}\right) ^{j}\right) \right) \left( x_{0}\right) \right\| _{\gamma }\nonumber \\{} & {} \qquad +\frac{\omega _{1}\left( f^{\left( m\right) },r\left( \left( {\widetilde{L}} _{n}\left( \left\| \cdot -x_{0}\right\| _{p}^{m+1}\right) \right) \left( x_{0}\right) \right) ^{\frac{1}{m+1}}\right) }{rm!}\left( \left( {\widetilde{L}}_{n}\left( \left\| \cdot -x_{0}\right\| _{p}^{m+1}\right) \right) \left( x_{0}\right) \right) ^{\left( \frac{m}{m+1}\right) }\nonumber \\{} & {} \qquad \times \left[ \frac{1}{\left( m+1\right) }+\frac{r}{2}+\frac{mr^{2}}{8}\right] ; \end{aligned}$$
  and
4. 58 $$\begin{aligned}{} & {} \left\| \left\| L_{n}\left( f\right) -f\right\| _{\gamma }\right\| _{\infty ,\prod _{i=1}^{N}\left[ a_{i},b_{i}\right] }\nonumber \\{} & {} \quad \le \sum _{j=1}^{m}\frac{1}{j!}\left\| \left\| \left( L_{n}\left( f^{\left( j\right) }\left( x_{0}\right) \left( \cdot -x_{0}\right) ^{j}\right) \right) \left( x_{0}\right) \right\| _{\gamma }\right\| _{\infty ,x_{0}\in \prod _{i=1}^{N}\left[ a_{i},b_{i}\right] }\nonumber \\{} & {} \qquad + \frac{\omega _{1}\left( f^{\left( m\right) },r\left\| \left( {\widetilde{L}} _{n}\left( \left\| \cdot -x_{0}\right\| _{p}^{m+1}\right) \right) \left( x_{0}\right) \right\| _{\infty ,x_{0}\in \prod _{i=1}^{N} \left[ a_{i},b_{i}\right] }^{\frac{1}{m+1}}\right) }{rm!}\nonumber \\{} & {} \qquad \times \left\| \left( {\widetilde{L}}_{n}\left( \left\| \cdot -x_{0}\right\| _{p}^{m+1}\right) \right) \left( x_{0}\right) \right\| _{\infty ,x_{0}\in \prod _{i=1}^{N}\left[ a_{i},b_{i}\right] }^{\left( \frac{m}{m+1} \right) } \nonumber \\{} & {} \qquad \times \left[ \frac{1}{\left( m+1\right) }+\frac{r}{2}+\frac{mr^{2}}{8}\right] . \end{aligned}$$

We need the following.

### Lemma 3.4

The function $\left( {\widetilde{L}}_{n}\left( \left\| \cdot -x_{0}\right\| _{p}^{m}\right) \right) \left( x_{0}\right)$ is continuous in $x_{0}\in \left( \prod _{i=1}^{N}\left[ a_{i},b_{i} \right] \right) ,$ $m\in {\mathbb {N}}.$

### Proof

By Lemma 10.3, [15, p. 272]. $$\square $$

### Remark 3.5

By Remark 10.4 [15, p. 273], we get that

59 $$\begin{aligned} \left\| \left( {\widetilde{L}}_{n}\left( \left\| \cdot -x_{0}\right\| _{p}^{k}\right) \right) \left( x_{0}\right) \right\| _{\infty ,x_{0}\in \prod _{i=1}^{N}\left[ a_{i},b_{i}\right] }\le \left\| \left( {\widetilde{L}}_{n}\left( \left\| \cdot -x_{0}\right\| _{p}^{m+1}\right) \right) \left( x_{0}\right) \right\| _{\infty ,x_{0}\in \prod _{i=1}^{N}\left[ a_{i},b_{i}\right] }^{\left( \frac{k}{m+1} \right) } \end{aligned}$$

for all $$k=1,\ldots ,m.$$ $$\square $$

We give the following.

### Corollary 3.6

(To Theorem 2.1, case of $$m=1)$$ Then

1. 60 $$\begin{aligned}{} & {} \left\| \left( L_{n}\left( f\right) \right) \left( x_{0}\right) -f\left( x_{0}\right) \right\| _{\gamma }\le \left\| \left( L_{n}\left( f^{\left( 1\right) }\left( x_{0}\right) \left( \cdot -x_{0}\right) \right) \right) \left( x_{0}\right) \right\| _{\gamma }\nonumber \\{} & {} \quad +\frac{1}{2r}\omega _{1}\left( f^{\left( 1\right) },r\left( \left( \widetilde{ L}_{n}\left( \left\| \cdot -x_{0}\right\| _{p}^{2}\right) \right) \left( x_{0}\right) \right) ^{\frac{1}{2}}\right) \left( \left( {\widetilde{L}} _{n}\left( \left\| \cdot -x_{0}\right\| _{p}^{2}\right) \right) \left( x_{0}\right) \right) ^{\frac{1}{2}}\nonumber \\{} & {} \quad \times \left[ 1+r+\frac{r^{2}}{4}\right] , \end{aligned}$$
  and
2. 61 $$\begin{aligned}{} & {} \left\| \left\| \left( L_{n}\left( f\right) \right) -f\right\| _{\gamma }\right\| _{\infty ,\prod _{i=1}^{N}\left[ a_{i},b_{i} \right] }\nonumber \\{} & {} \quad \le \left\| \left\| \left( L_{n}\left( f^{\left( 1\right) }\left( x_{0}\right) \left( \cdot -x_{0}\right) \right) \right) \left( x_{0}\right) \right\| _{\gamma }\right\| _{\infty ,x_{0}\in \prod _{i=1}^{N} \left[ a_{i},b_{i}\right] }\nonumber \\{} & {} \qquad + \frac{1}{2r}\omega _{1}\left( f^{\left( 1\right) },r\left\| \left( {\widetilde{L}}_{n}\left( \left\| \cdot -x_{0}\right\| _{p}^{2}\right) \right) \left( x_{0}\right) \right\| _{\infty ,x_{0}\in \prod _{i=1}^{N}\left[ a_{i},b_{i}\right] }^{\frac{1}{2}}\right) \nonumber \\{} & {} \qquad \times \left\| \left( {\widetilde{L}}_{n}\left( \left\| \cdot -x_{0}\right\| _{p}^{2}\right) \right) \left( x_{0}\right) \right\| _{\infty ,x_{0}\in \prod _{i=1}^{N}\left[ a_{i},b_{i}\right] }^{\frac{1}{2}}\left[ 1+r+ \frac{r^{2}}{4}\right] , \end{aligned}$$

$$r>0.$$

We make the following.

### Remark 3.7

We estimate $$0<\alpha <1,$$ $$\mu >0,$$ $$m,n\in {\mathbb {N}} :n^{1-\alpha }>2,$$

62 $$\begin{aligned}{} & {} {\widetilde{L}}_{n}\left( \left\| \cdot -x_{0}\right\| _{\infty }^{m+1}\right) \left( x_{0}\right) =\frac{\sum _{k=\left\lceil na\right\rceil }^{\left\lfloor nb\right\rfloor }\left\| \frac{k}{n}-x_{0}\right\| _{\infty }^{m+1}Z\left( nx_{0}-k\right) }{\sum _{k=\left\lceil na\right\rceil }^{\left\lfloor nb\right\rfloor }Z\left( nx_{0}-k\right) }\nonumber \\{} & {} \quad \overset{(19)}{<} \left( \frac{4\left( 1+{\textrm{e}}^{-2\mu }\right) }{1-{\textrm{e}}^{-2\mu }}\right) ^{N}\sum _{k=\left\lceil na\right\rceil }^{\left\lfloor nb\right\rfloor }\left\| \frac{k}{n}-x_{0}\right\| _{\infty }^{m+1}Z\left( nx_{0}-k\right) \end{aligned}$$

63 $$\begin{aligned}{} & {} \quad =\left( \frac{4\left( 1+{\textrm{e}}^{-2\mu }\right) }{1-{\textrm{e}}^{-2\mu }}\right) ^{N}\left\{ \sum _{\left\{ \begin{array}{c} k=\left\lceil na\right\rceil \\ :\left\| \frac{k}{n}-x_{0}\right\| _{\infty }\le \frac{1}{n^{\alpha }} \end{array} \right. }^{\left\lfloor nb\right\rfloor }\left\| \frac{k}{n} -x_{0}\right\| _{\infty }^{m+1}Z\left( nx_{0}-k\right) \right. \nonumber \\{} & {} \qquad + \left. \sum _{\left\{ \begin{array}{c} k=\left\lceil na\right\rceil \\ :\left\| \frac{k}{n}-x_{0}\right\| _{\infty }>\frac{1}{n^{\alpha }} \end{array} \right. }^{\left\lfloor nb\right\rfloor }\left\| \frac{k}{n} -x_{0}\right\| _{\infty }^{m+1}Z\left( nx_{0}-k\right) \right\} \nonumber \\{} & {} \quad \overset{(20)}{\le } \left( \frac{4\left( 1+{\textrm{e}}^{-2\mu }\right) }{1-{\textrm{e}}^{-2\mu }}\right) ^{N}\left\{ \frac{1}{n^{\alpha \left( m+1\right) }}+\frac{\left\| b-a\right\| _{\infty }^{m+1}}{{\textrm{e}}^{\mu \left( n^{1-\beta }-2\right) }}\right\} \end{aligned}$$

(where $$b-a=\left( b_{1}-a_{1},\ldots ,b_{N}-a_{N}\right) $$).

We have proved that $(\forall$ $x_{0}\in \prod _{i=1}^{N}\left[ a_{i},b_{i}\right] )$

64 $$\begin{aligned} {\widetilde{L}}_{n}\left( \left\| \cdot -x_{0}\right\| _{\infty }^{m+1}\right) \left( x_{0}\right) <\left( \frac{4\left( 1+{\textrm{e}}^{-2\mu }\right) }{1-{\textrm{e}}^{-2\mu }}\right) ^{N}\left\{ \frac{1}{n^{\alpha \left( m+1\right) }}+ \frac{\left\| b-a\right\| _{\infty }^{m+1}}{{\textrm{e}}^{\mu \left( n^{1-\beta }-2\right) }}\right\} =:\Lambda _{1}\left( n\right) \end{aligned}$$

$$(0<\alpha <1,$$ $$m,n\in {\mathbb {N}}:n^{1-\alpha }>2,$$ $$\mu >0).$$

And, consequently, it holds

65 $$\begin{aligned}{} & {} \left\| {\widetilde{L}}_{n}\left( \left\| \cdot -x_{0}\right\| _{\infty }^{m+1}\right) \left( x_{0}\right) \right\| _{\infty ,x_{0}\in \prod _{i=1}^{N}\left[ a_{i},b_{i}\right] }\nonumber \\{} & {} \quad <\left( \frac{4\left( 1+{\textrm{e}}^{-2\mu }\right) }{1-{\textrm{e}}^{-2\mu }}\right) ^{N}\left\{ \frac{1}{n^{\alpha \left( m+1\right) }}+\frac{\left\| b-a\right\| _{\infty }^{m+1}}{{\textrm{e}}^{\mu \left( n^{1-\beta }-2\right) }}\right\} =\Lambda _{1}\left( n\right) \rightarrow 0,\quad \text {as }n\rightarrow +\infty . \end{aligned}$$

Therefore, we have that $\Lambda _{1}\left( n\right) \rightarrow 0,$ as $n\rightarrow +\infty .$ Thus, when $p\in \left[ 1,\infty \right] ,$ from Theorem 2.1, we have the convergence to zero in the right-hand sides of parts (1), (2).

Next, we estimate $\left\| \left( {\widetilde{L}}_{n}\left( f^{\left( j\right) }\left( x_{0}\right) \left( \cdot -x_{0}\right) ^{j}\right) \right) \left( x_{0}\right) \right\| _{\gamma }.$

We have that

66 $$\begin{aligned} \left( {\widetilde{L}}_{n}\left( f^{\left( j\right) }\left( x_{0}\right) \left( \cdot -x_{0}\right) ^{j}\right) \right) \left( x_{0}\right) =\frac{ \sum _{k=\left\lceil na\right\rceil }^{\left\lfloor nb\right\rfloor }f^{\left( j\right) }\left( x_{0}\right) \left( \frac{k}{n}-x_{0}\right) ^{j}Z\left( nx_{0}-k\right) }{\sum _{k=\left\lceil na\right\rceil }^{\left\lfloor nb\right\rfloor }Z\left( nx_{0}-k\right) }. \end{aligned}$$

When $p=\infty ,$ $j=1,\ldots ,m,$ we obtain

67 $$\begin{aligned} \left\| f^{\left( j\right) }\left( x_{0}\right) \left( \frac{k}{n} -x_{0}\right) ^{j}\right\| _{\gamma }\le \left\| f^{\left( j\right) }\left( x_{0}\right) \right\| \left\| \frac{k}{n}-x_{0}\right\| _{\infty }^{j}. \end{aligned}$$

We further have that

68 $$\begin{aligned}{} & {} \left\| \left( {\widetilde{L}}_{n}\left( f^{\left( j\right) }\left( x_{0}\right) \left( \cdot -x_{0}\right) ^{j}\right) \right) \left( x_{0}\right) \right\| _{\gamma }\nonumber \\{} & {} \quad \overset{(19)}{<} \left( \frac{4\left( 1+{\textrm{e}}^{-2\mu }\right) }{1-{\textrm{e}}^{-2\mu }}\right) ^{N}\left( \sum _{k=\left\lceil na\right\rceil }^{\left\lfloor nb\right\rfloor }\left\| f^{\left( j\right) }\left( x_{0}\right) \left( \frac{k}{n} -x_{0}\right) ^{j}\right\| _{\gamma }Z\left( nx_{0}-k\right) \right) \nonumber \\{} & {} \quad \le \left( \frac{4\left( 1+{\textrm{e}}^{-2\mu }\right) }{1-{\textrm{e}}^{-2\mu }}\right) ^{N}\left( \sum _{k=\left\lceil na\right\rceil }^{\left\lfloor nb\right\rfloor }\left\| f^{\left( j\right) }\left( x_{0}\right) \right\| \left\| \frac{k}{n}-x_{0}\right\| _{\infty }^{j}Z\left( nx_{0}-k\right) \right) \end{aligned}$$

69 $$\begin{aligned}{} & {} \quad = \left( \frac{4\left( 1+{\textrm{e}}^{-2\mu }\right) }{1-{\textrm{e}}^{-2\mu }}\right) ^{N}\left\| f^{\left( j\right) }\left( x_{0}\right) \right\| \left( \sum _{k=\left\lceil na\right\rceil }^{\left\lfloor nb\right\rfloor }\left\| \frac{k}{n}-x_{0}\right\| _{\infty }^{j}Z\left( nx_{0}-k\right) \right) \nonumber \\{} & {} \quad = \left( \frac{4\left( 1+{\textrm{e}}^{-2\mu }\right) }{1-{\textrm{e}}^{-2\mu }}\right) ^{N}\left\| f^{\left( j\right) }\left( x_{0}\right) \right\| \left\{ \sum _{\left\{ \begin{array}{c} k=\left\lceil na\right\rceil \\ :\left\| \frac{k}{n}-x_{0}\right\| _{\infty }\le \frac{1}{n^{\alpha }} \end{array} \right. }^{\left\lfloor nb\right\rfloor }\left\| \frac{k}{n} -x_{0}\right\| _{\infty }^{j}Z\left( nx_{0}-k\right) \right. \nonumber \\{} & {} \qquad \left. +\sum _{\left\{ \begin{array}{c} k=\left\lceil na\right\rceil \\ :\left\| \frac{k}{n}-x_{0}\right\| _{\infty }>\frac{1}{n^{\alpha }} \end{array} \right. }^{\left\lfloor nb\right\rfloor }\left\| \frac{k}{n} -x_{0}\right\| _{\infty }^{j}Z\left( nx_{0}-k\right) \right\} \nonumber \\{} & {} \quad \overset{(20)}{\le } \left( \frac{4\left( 1+{\textrm{e}}^{-2\mu }\right) }{1-{\textrm{e}}^{-2\mu }}\right) ^{N}\left\| f^{\left( j\right) }\left( x_{0}\right) \right\| \left\{ \frac{1}{n^{\alpha j}}+\frac{\left\| b-a\right\| _{\infty }^{j}}{ {\textrm{e}}^{\mu \left( n^{1-\beta }-2\right) }}\right\} \rightarrow 0, \quad \text {as } n\rightarrow \infty . \end{aligned}$$

That is

$$\begin{aligned} \left\| \left( {\widetilde{L}}_{n}\left( f^{\left( j\right) }\left( x_{0}\right) \left( \cdot -x_{0}\right) ^{j}\right) \right) \left( x_{0}\right) \right\| _{\gamma }\rightarrow 0, \quad \text {as }n\rightarrow \infty . \end{aligned}$$

Therefore, when $p=\infty ,$ for $j=1,\ldots ,m,$ we have proved

70 $$\begin{aligned}{} & {} \left\| \left( {\widetilde{L}}_{n}\left( f^{\left( j\right) }\left( x_{0}\right) \left( \cdot -x_{0}\right) ^{j}\right) \right) \left( x_{0}\right) \right\| _{\gamma }\nonumber \\{} & {} \quad< \left( \frac{4\left( 1+{\textrm{e}}^{-2\mu }\right) }{1-{\textrm{e}}^{-2\mu }}\right) ^{N}\left\| f^{\left( j\right) }\left( x_{0}\right) \right\| \left\{ \frac{1}{n^{\alpha j}}+\frac{\left\| b-a\right\| _{\infty }^{j}}{ {\textrm{e}}^{\mu \left( n^{1-\beta }-2\right) }}\right\} \nonumber \\{} & {} \quad \le \left( \frac{4\left( 1+{\textrm{e}}^{-2\mu }\right) }{1-{\textrm{e}}^{-2\mu }}\right) ^{N}\left\| f^{\left( j\right) }\left( x_{0}\right) \right\| _{\infty }\left\{ \frac{1}{n^{\alpha j}}+\frac{\left\| b-a\right\| _{\infty }^{j}}{{\textrm{e}}^{\mu \left( n^{1-\beta }-2\right) }}\right\} =:\Lambda _{2j}\left( n\right) <\infty , \end{aligned}$$

and converges to zero, as $n\rightarrow \infty .$

We conclude the following:

In Theorem 2.1, the right-hand sides of (57) and (58) converge to zero as $n\rightarrow \infty ,$ for any $p\in \left[ 1,\infty \right] .$

Also in Corollary 3.6, the right-hand sides of (60) and (61) converge to zero as $n\rightarrow \infty ,$ for any $p\in \left[ 1,\infty \right] .$

### Conclusion 3.8

We have proved that the left-hand sides of (55), (56), (57), (58), and (60), (61) converge to zero as $n\rightarrow \infty ,$ for $p\in \left[ 1,\infty \right] .$ Consequently,  $L_{n}\rightarrow I$ (unit operator) pointwise and uniformly, as $n\rightarrow \infty ,$ where $p\in \left[ 1,\infty \right] .$ In the presence of initial conditions,  we achieve a higher speed of convergence;  see (56). Higher speed of convergence happens also to the left-hand side of (55).

We further give the following:

### Corollary 3.9

(To Theorem 2.1) Let *O* open subset of $\left( {\mathbb {R}} ^{N},\left\| \cdot \right\| _{\infty }\right) ,$ such that $\prod _{i=1}^{N}\left[ a_{i},b_{i}\right] \subset O\subseteq {\mathbb {R}} ^{N},$ and let $\left( X,\left\| \cdot \right\| _{\gamma }\right)$ be a general Banach space. Let $m\in {\mathbb {N}}$ and $f\in C^{m}\left( O,X\right) ,$ the space of *m*-times continuously Fréchet differentiable functions from *O* into *X*. We study the approximation of $f|_{\prod _{i=1}^{N}\left[ a_{i},b_{i}\right] }.$ Let $x_{0}\in \left( \prod _{i=1}^{N}\left[ a_{i},b_{i}\right] \right)$ and $r>0.$ Here, $\Lambda _{1}\left( n\right)$ as in (65) and $\Lambda _{2j}\left( n\right)$ as in (70), where $n\in {\mathbb {N}}:n^{1-\alpha }>2,$ $0<\alpha <1,$ $\mu >0,$ $j=1,\ldots ,m.$ Then

1. 71 $$\begin{aligned}{} & {} \left\| \left( L_{n}\left( f\right) \right) \left( x_{0}\right) -\sum _{j=0}^{m}\frac{1}{j!}\left( L_{n}\left( f^{\left( j\right) }\left( x_{0}\right) \left( \cdot -x_{0}\right) ^{j}\right) \right) \left( x_{0}\right) \right\| _{\gamma }\nonumber \\{} & {} \quad \le \frac{\omega _{1}\left( f^{\left( m\right) },r\left( \Lambda _{1}\left( n\right) \right) ^{\frac{1}{m+1}}\right) }{rm!}\left( \Lambda _{1}\left( n\right) \right) ^{\left( \frac{m}{m+1}\right) }\left[ \frac{1}{\left( m+1\right) }+\frac{r}{2}+\frac{mr^{2}}{8}\right] ; \end{aligned}$$
2. in addition,  if $f^{\left( j\right) }\left( x_{0}\right) =0,$ $j=1,\ldots ,m,$ we have
  72 $$\begin{aligned}{} & {} \left\| \left( L_{n}\left( f\right) \right) \left( x_{0}\right) -f\left( x_{0}\right) \right\| _{\gamma }\nonumber \\{} & {} \quad \le \frac{\omega _{1}\left( f^{\left( m\right) },r\left( \Lambda _{1}\left( n\right) \right) ^{\frac{1}{m+1}}\right) }{rm!}\left( \Lambda _{1}\left( n\right) \right) ^{\left( \frac{m}{m+1}\right) }\left[ \frac{1}{\left( m+1\right) }+\frac{r}{2}+\frac{mr^{2}}{8}\right] ; \end{aligned}$$
3. 73 $$\begin{aligned}{} & {} \left\| \left\| L_{n}\left( f\right) -f\right\| _{\gamma }\right\| _{\infty ,\prod _{i=1}^{N}\left[ a_{i},b_{i}\right] }\le \sum _{j=1}^{m}\frac{\Lambda _{2j}\left( n\right) }{j!}\nonumber \\{} & {} \quad + \frac{\omega _{1}\left( f^{\left( m\right) },r\left( \Lambda _{1}\left( n\right) \right) ^{\frac{1}{m+1}}\right) }{rm!}\left( \Lambda _{1}\left( n\right) \right) ^{\left( \frac{m}{m+1}\right) } \nonumber \\{} & {} \quad \times \left[ \frac{1}{\left( m+1\right) }+\frac{r}{2}+\frac{mr^{2}}{8}\right] =:\Lambda _{3}\left( n\right) \rightarrow 0, \quad \text {as }n\rightarrow \infty . \end{aligned}$$

We continue with the following.

### Theorem 3.10

Let $f\in C_{B}\left( {\mathbb {R}}^{N},X\right) ,$ $0<\beta <1,$ $\mu >0,$ $x\in {\mathbb {R}}^{N},N,n\in {\mathbb {N}}$ with $n^{1-\beta }>2,$ $\omega _{1}$ is for $p=\infty .$ Then

1. 74 $$\begin{aligned} \left\| B_{n}\left( f,x\right) -f\left( x\right) \right\| _{\gamma }\le \omega _{1}\left( f,\frac{1}{n^{\beta }}\right) +\frac{2\left\| \left\| f\right\| _{\gamma }\right\| _{\infty }}{{\textrm{e}}^{\mu \left( n^{1-\beta }-2\right) }}=:\lambda _{2}\left( n\right) ; \end{aligned}$$
2. 75 $$\begin{aligned} \left\| \left\| B_{n}\left( f\right) -f\right\| _{\gamma }\right\| _{\infty }\le \lambda _{2}\left( n\right) . \end{aligned}$$
  Given that $f\in \left( C_{U}\left( {\mathbb {R}}^{N},X\right) \cap C_{B}\left( {\mathbb {R}}^{N},X\right) \right) ,$ we obtain $\lim _{n\rightarrow \infty } B_{n}\left( f\right) =f,$ uniformly. The speed of convergence above is $\max \left( \frac{1}{n^{\beta }},\frac{2}{{\textrm{e}}^{\left( n^{1-\beta }-2\right) }}\right) =\frac{1}{n^{\beta }}.$

### Proof

We have that

76 $$\begin{aligned}{} & {} B_{n}\left( f,x\right) -f\left( x\right) \overset{(13)}{=} \sum _{k=-\infty }^{\infty }f\left( \frac{k}{n}\right) Z\left( nx-k\right) -f\left( x\right) \sum _{k=-\infty }^{\infty }Z\left( nx-k\right) \nonumber \\{} & {} \quad \qquad \qquad \qquad \quad \qquad = \sum _{k=-\infty }^{\infty }\left( f\left( \frac{k}{n}\right) -f\left( x\right) \right) Z\left( nx-k\right) . \end{aligned}$$

Hence

77 $$\begin{aligned}{} & {} \left\| B_{n}\left( f,x\right) -f\left( x\right) \right\| _{\gamma }\le \sum _{k=-\infty }^{\infty }\left\| f\left( \frac{k}{n}\right) -f\left( x\right) \right\| _{\gamma }Z\left( nx-k\right) \nonumber \\{} & {} \quad = \sum _{\left\{ \begin{array}{c} k=-\infty \\ \left\| \frac{k}{n}-x\right\| _{\infty }\le \frac{1}{n^{\beta }} \end{array} \right. }^{\infty }\left\| f\left( \frac{k}{n}\right) -f\left( x\right) \right\| _{\gamma }Z\left( nx-k\right) \nonumber \\{} & {} \qquad + \sum _{\left\{ \begin{array}{c} k=-\infty \\ \left\| \frac{k}{n}-x\right\| _{\infty }>\frac{1}{n^{\beta }} \end{array} \right. }^{\infty }\left\| f\left( \frac{k}{n}\right) -f\left( x\right) \right\| _{\gamma }Z\left( nx-k\right) \nonumber \\{} & {} \quad \overset{(13)}{\le } \omega _{1}\left( f,\frac{1}{n^{\beta }}\right) +2\left\| \left\| f\right\| _{\gamma }\right\| _{\infty }\sum _{\left\{ \begin{array}{c} k=-\infty \\ \left\| \frac{k}{n}-x\right\| _{\infty }>\frac{1}{n^{\beta }} \end{array} \right. }^{\infty }Z\left( nx-k\right) \nonumber \\{} & {} \quad \overset{(20)}{\le } \omega _{1}\left( f,\frac{1}{n^{\beta }}\right) +\frac{2\left\| \left\| f\right\| _{\gamma }\right\| _{\infty }}{{\textrm{e}}^{\mu \left( n^{1-\beta }-2\right) }}, \end{aligned}$$

proving the claim. $\square$

We give the following.

### Theorem 3.11

Let $f\in C_{B}\left( {\mathbb {R}}^{N},X\right) ,$ $0<\beta <1,$ $x\in {\mathbb {R}}^{N},$ $\mu >0,N,n\in {\mathbb {N}}$ with $n^{1-\beta }>2,$ $\omega _{1}$ is for $p=\infty .$ Then

1. 78 $$\begin{aligned} \left\| C_{n}\left( f,x\right) -f\left( x\right) \right\| _{\gamma }\le \omega _{1}\left( f,\frac{1}{n}+\frac{1}{n^{\beta }}\right) +\frac{ 2\left\| \left\| f\right\| _{\gamma }\right\| _{\infty }}{{\textrm{e}}^{\mu \left( n^{1-\beta }-2\right) }}=:\lambda _{3}\left( n\right) ; \end{aligned}$$
2. 79 $$\begin{aligned} \left\| \left\| C_{n}\left( f\right) -f\right\| _{\gamma }\right\| _{\infty }\le \lambda _{3}\left( n\right) . \end{aligned}$$
  Given that $f\in \left( C_{U}\left( {\mathbb {R}}^{N},X\right) \cap C_{B}\left( {\mathbb {R}}^{N},X\right) \right) ,$ we obtain $\lim _{n\rightarrow \infty } C_{n}\left( f\right) =f,$ uniformly.

### Proof

We notice that

80 $$\begin{aligned}{} & {} \int _{\frac{k}{n}}^{\frac{k+1}{n}}f\left( t\right) {\textrm{d}}t=\int _{\frac{k_{1}}{n} }^{\frac{k_{1}+1}{n}}\int _{\frac{k_{2}}{n}}^{\frac{k_{2}+1}{n}}\cdots \int _{ \frac{k_{N}}{n}}^{\frac{k_{N}+1}{n}}f\left( t_{1},t_{2},\ldots ,t_{N}\right) {\textrm{d}}t_{1}{\textrm{d}}t_{2}\ldots {\textrm{d}}t_{N}\nonumber \\{} & {} \quad = \int _{0}^{\frac{1}{n}}\int _{0}^{\frac{1}{n}}\cdots \int _{0}^{\frac{1}{n}}f\left( t_{1}+\frac{k_{1}}{n},t_{2}+\frac{k_{2}}{n},\ldots ,t_{N}+\frac{k_{N}}{n}\right) {\textrm{d}}t_{1}\cdots {\textrm{d}}t_{N}=\int _{0}^{\frac{1}{n}}f\left( t+\frac{k}{n}\right) {\textrm{d}}t. \end{aligned}$$

Thus, it holds (by (35))

81 $$\begin{aligned} C_{n}\left( f,x\right) =\sum _{k=-\infty }^{\infty }\left( n^{N}\int _{0}^{ \frac{1}{n}}f\left( t+\frac{k}{n}\right) {\textrm{d}}t\right) Z\left( nx-k\right) . \end{aligned}$$

We observe that

82 $$\begin{aligned}{} & {} \left\| C_{n}\left( f,x\right) -f\left( x\right) \right\| _{\gamma }\nonumber \\{} & {} \quad = \left\| \sum _{k=-\infty }^{\infty }\left( n^{N}\int _{0}^{\frac{1}{n} }f\left( t+\frac{k}{n}\right) {\textrm{d}}t\right) Z\left( nx-k\right) -\sum _{k=-\infty }^{\infty }f\left( x\right) Z\left( nx-k\right) \right\| _{\gamma }\nonumber \\{} & {} \quad = \left\| \sum _{k=-\infty }^{\infty }\left( \left( n^{N}\int _{0}^{\frac{1}{n }}f\left( t+\frac{k}{n}\right) {\textrm{d}}t\right) -f\left( x\right) \right) Z\left( nx-k\right) \right\| _{\gamma }\nonumber \\{} & {} \quad = \left\| \sum _{k=-\infty }^{\infty }\left( n^{N}\int _{0}^{\frac{1}{n} }\left( f\left( t+\frac{k}{n}\right) -f\left( x\right) \right) {\textrm{d}}t\right) Z\left( nx-k\right) \right\| _{\gamma }\end{aligned}$$

83 $$\begin{aligned}{} & {} \quad \le \sum _{k=-\infty }^{\infty }\left( n^{N}\int _{0}^{\frac{1}{n}}\left\| f\left( t+\frac{k}{n}\right) -f\left( x\right) \right\| _{\gamma }{\textrm{d}}t\right) Z\left( nx-k\right) \nonumber \\{} & {} \quad = \sum _{\left\{ \begin{array}{c} k=-\infty \\ \left\| \frac{k}{n}-x\right\| _{\infty }\le \frac{1}{n^{\beta }} \end{array} \right. }^{\infty }\left( n^{N}\int _{0}^{\frac{1}{n}}\left\| f\left( t+ \frac{k}{n}\right) -f\left( x\right) \right\| _{\gamma }{\textrm{d}}t\right) Z\left( nx-k\right) \nonumber \\{} & {} \qquad + \sum _{\left\{ \begin{array}{c} k=-\infty \\ \left\| \frac{k}{n}-x\right\| _{\infty }>\frac{1}{n^{\beta }} \end{array} \right. }^{\infty }\left( n^{N}\int _{0}^{\frac{1}{n}}\left\| f\left( t+ \frac{k}{n}\right) -f\left( x\right) \right\| _{\gamma }{\textrm{d}}t\right) Z\left( nx-k\right) \nonumber \\{} & {} \quad \le \sum _{\left\{ \begin{array}{c} k=-\infty \\ \left\| \frac{k}{n}-x\right\| _{\infty }\le \frac{1}{n^{\beta }} \end{array} \right. }^{\infty }\left( n^{N}\int _{0}^{\frac{1}{n}}\omega _{1}\left( f,\left\| t\right\| _{\infty }+\left\| \frac{k}{n}-x\right\| _{\infty }\right) {\textrm{d}}t\right) Z\left( nx-k\right) \nonumber \\{} & {} \qquad + 2\left\| \left\| f\right\| _{\gamma }\right\| _{\infty }\left( \sum _{\left\{ \begin{array}{c} k=-\infty \\ \left\| \frac{k}{n}-x\right\| _{\infty }>\frac{1}{n^{\beta }} \end{array} \right. }^{\infty }Z\left( \left| nx-k\right| \right) \right) \nonumber \\{} & {} \quad \le \omega _{1}\left( f,\frac{1}{n}+\frac{1}{n^{\beta }}\right) +\frac{ 2\left\| \left\| f\right\| _{\gamma }\right\| _{\infty }}{{\textrm{e}}^{\mu \left( n^{1-\beta }-2\right) }}, \end{aligned}$$

proving the claim. $\square$

We also present the following.

### Theorem 3.12

Let $f\in C_{B}\left( {\mathbb {R}}^{N},X\right) ,$ $0<\beta <1,$ $x\in {\mathbb {R}}^{N},$ $\mu >0,N,n\in {\mathbb {N}}$ with $n^{1-\beta }>2,$ $\omega _{1}$ is for $p=\infty .$ Then

1. 84 $$\begin{aligned} \left\| D_{n}\left( f,x\right) -f\left( x\right) \right\| _{\gamma }\le \omega _{1}\left( f,\frac{1}{n}+\frac{1}{n^{\beta }}\right) +\frac{ 2\left\| \left\| f\right\| _{\gamma }\right\| _{\infty }}{{\textrm{e}}^{\mu \left( n^{1-\beta }-2\right) }}=:\lambda _{4}\left( n\right) ; \end{aligned}$$
2. 85 $$\begin{aligned} \left\| \left\| D_{n}\left( f\right) -f\right\| _{\gamma }\right\| _{\infty }\le \lambda _{4}\left( n\right) . \end{aligned}$$

Given that $f\in \left( C_{U}\left( {\mathbb {R}}^{N},X\right) \cap C_{B}\left( {\mathbb {R}}^{N},X\right) \right) ,$ we obtain $\lim _{n\rightarrow \infty } D_{n}\left( f\right) =f,$ uniformly.

### Proof

We have that [by (37)]

$$\begin{aligned}{} & {} \left\| D_{n}\left( f,x\right) -f\left( x\right) \right\| _{\gamma }=\left\| \sum _{k=-\infty }^{\infty }\delta _{nk}\left( f\right) Z\left( nx-k\right) -\sum _{k=-\infty }^{\infty }f\left( x\right) Z\left( nx-k\right) \right\| _{\gamma }\\{} & {} \quad = \left\| \sum _{k=-\infty }^{\infty }\left( \delta _{nk}\left( f\right) -f\left( x\right) \right) Z\left( nx-k\right) \right\| _{\gamma }=\left\| \sum _{k=-\infty }^{\infty }w_{r}\left( f\left( \frac{k}{n}+ \frac{r}{n\theta }\right) -f\left( x\right) \right) Z\left( nx-k\right) \right\| _{\gamma }\\{} & {} \quad \le \sum _{k=-\infty }^{\infty }\left( \sum _{r=0}^{\theta }w_{r}\left\| f\left( \frac{k}{n}+\frac{r}{n\theta }\right) -f\left( x\right) \right\| _{\gamma }\right) Z\left( nx-k\right) \\{} & {} \quad = \sum _{\left\{ \begin{array}{c} k=-\infty \\ \left\| \frac{k}{n}-x\right\| _{\infty }\le \frac{1}{n^{\beta }} \end{array} \right. }^{\infty }\left( \sum _{r=0}^{\theta }w_{r}\left\| f\left( \frac{k }{n}+\frac{r}{n\theta }\right) -f\left( x\right) \right\| _{\gamma }\right) Z\left( nx-k\right) \\{} & {} \qquad + \sum _{\left\{ \begin{array}{c} k=-\infty \\ \left\| \frac{k}{n}-x\right\| _{\infty }>\frac{1}{n^{\beta }} \end{array} \right. }^{\infty }\left( \sum _{r=0}^{\theta }w_{r}\left\| f\left( \frac{k }{n}+\frac{r}{n\theta }\right) -f\left( x\right) \right\| _{\gamma }\right) Z\left( nx-k\right) \\{} & {} \quad \le \sum _{\left\{ \begin{array}{c} k=-\infty \\ \left\| \frac{k}{n}-x\right\| _{\infty }\le \frac{1}{n^{\beta }} \end{array} \right. }^{\infty }\left( \sum _{r=0}^{\theta }w_{r}\left\| f\left( \frac{k }{n}+\frac{r}{n\theta }\right) -f\left( x\right) \right\| _{\gamma }\right) Z\left( nx-k\right) \\{} & {} \qquad + 2\left\| \left\| f\right\| _{\gamma }\right\| _{\infty }\left( \sum _{\left\{ \begin{array}{c} k=-\infty \\ \left\| \frac{k}{n}-x\right\| _{\infty }>\frac{1}{n^{\beta }} \end{array} \right. }^{\infty }\left( Z\left( nx-k\right) \right) \right) \\{} & {} \quad \le \omega _{1}\left( f,\frac{1}{n}+\frac{1}{n^{\beta }}\right) +\frac{ 2\left\| \left\| f\right\| _{\gamma }\right\| _{\infty }}{{\textrm{e}}^{\mu \left( n^{1-\beta }-2\right) }}=\lambda _{4}\left( n\right) , \end{aligned}$$

proving the claim. $\square$

We make the following.

### Definition 3.13

Let $f\in C_{B}\left( {\mathbb {R}}^{N},X\right) ,$ $N\in {\mathbb {N}},$ where $\left( X,\left\| \cdot \right\| _{\gamma }\right)$ is a Banach space. We define the general neural network operator

86 $$\begin{aligned}{} & {} F_{n}\left( f,x\right) :=\sum _{k=-\infty }^{\infty }l_{nk}\left( f\right) Z\left( nx-k\right) \nonumber \\{} & {} \quad = \left\{ \begin{array}{ll} B_{n}\left( f,x\right) , &{}\quad \text {if }l_{nk}\left( f\right) =f\left( \frac{k}{ n}\right) , \\ C_{n}\left( f,x\right) , &{}\quad \text {if }l_{nk}\left( f\right) =n^{N}\int _{\frac{ k}{n}}^{\frac{k+1}{n}}f\left( t\right) {\textrm{d}}t, \\ D_{n}\left( f,x\right) , &{}\quad \text {if }l_{nk}\left( f\right) =\delta _{nk}\left( f\right) . \end{array} \right. \end{aligned}$$

Clearly, $l_{nk}\left( f\right)$ is an *X*-valued bounded linear functional, such that $\left\| l_{nk}\left( f\right) \right\| _{\gamma }\le \left\| \left\| f\right\| _{\gamma }\right\| _{\infty }.$

Hence, $F_{n}\left( f\right)$ is a bounded linear operator with $\left\| \left\| F_{n}\left( f\right) \right\| _{\gamma }\right\| _{\infty }\le \left\| \left\| f\right\| _{\gamma }\right\| _{\infty }.$

We need the following.

### Theorem 3.14

Let $$f\in C_{B}\left( {\mathbb {R}}^{N},X\right) ,$$ $$N\ge 1.$$ Then,  $$F_{n}\left( f\right) \in C_{B}\left( {\mathbb {R}}^{N},X\right) .$$

### Proof

Lengthy and similar to the proof of Theorem 10 of [18], as such is omitted. $\square$

### Remark 3.15

By (22), it is obvious that $\left\| \left\| L_{n}\left( f\right) \right\| _{\gamma }\right\| _{\infty }\le \!\left\| \left\| f\right\| _{\gamma }\right\| _{\infty }\!<\!\infty$, and $L_{n}\!\left( f\right) \!\in \! C\left( \prod _{i=1}^{N}\left[ a_{i},b_{i}\right] ,X\right) ,$ given that $f\in C\left( \prod _{i=1}^{N}\left[ a_{i},b_{i}\right] ,X\right) .$

Call $K_{n}$ any of the operators $L_{n},B_{n},C_{n},D_{n}.$

Clearly, then

87 $$\begin{aligned} \left\| \left\| K_{n}^{2}\left( f\right) \right\| _{\gamma }\right\| _{\infty }=\left\| \left\| K_{n}\left( K_{n}\left( f\right) \right) \right\| _{\gamma }\right\| _{\infty }\le \left\| \left\| K_{n}\left( f\right) \right\| _{\gamma }\right\| _{\infty }\le \left\| \left\| f\right\| _{\gamma }\right\| _{\infty }, \end{aligned}$$

etc.

Therefore, we get

88 $$\begin{aligned} \left\| \left\| K_{n}^{k}\left( f\right) \right\| _{\gamma }\right\| _{\infty }\le \left\| \left\| f\right\| _{\gamma }\right\| _{\infty }, \quad \forall ~ k\in {\mathbb {N}}, \end{aligned}$$

the contraction property.

Also, we see that

89 $$\begin{aligned} \left\| \left\| K_{n}^{k}\left( f\right) \right\| _{\gamma }\right\| _{\infty }\le \left\| \left\| K_{n}^{k-1}\left( f\right) \right\| _{\gamma }\right\| _{\infty }\le \cdots \le \left\| \left\| K_{n}\left( f\right) \right\| _{\gamma }\right\| _{\infty }\le \left\| \left\| f\right\| _{\gamma }\right\| _{\infty }. \end{aligned}$$

Here, $K_{n}^{k}$ are bounded linear operators.

### Notation 3.16

Here,  $N\in {\mathbb {N}},$ $0<\beta <1.$ Denote by

90 $$\begin{aligned}{} & {} c_{N}:=\left\{ \begin{array}{ll} \left( \frac{4\left( 1+{\textrm{e}}^{-2\mu }\right) }{1-{\textrm{e}}^{-2\mu }}\right) ^{N}, &{}\quad \text {if }K_{n}=L_{n}, \\ 1, &{}\quad \text {if }K_{n}=B_{n},C_{n},D_{n}, \end{array} \right. \end{aligned}$$

91 $$\begin{aligned}{} & {} \Lambda \left( n\right) :=\left\{ \begin{array}{ll} \frac{1}{n^{\beta }}, &{}\quad \text {if }K_{n}=L_{n}, B_{n}, \\ \frac{1}{n}+\frac{1}{n^{\beta }}, &{}\quad \text {if }K_{n}=C_{n},D_{n}, \end{array} \right. \end{aligned}$$

92 $$\begin{aligned}{} & {} \Omega :=\left\{ \begin{array}{ll} C\left( \prod _{i=1}^{N}\left[ a_{i},b_{i}\right] ,X\right) ,&{}\quad \text {if }K_{n}=L_{n}, \\ C_{B}\left( {\mathbb {R}}^{N},X\right) , &{}\quad \text {if }K_{n}=B_{n},C_{n},D_{n}, \end{array} \right. \end{aligned}$$

and

93 $$\begin{aligned} Y:=\left\{ \begin{array}{ll} \prod _{i=1}^{N}\left[ a_{i},b_{i}\right] , &{}\quad \text {if }K_{n}=L_{n}, \\ {\mathbb {R}}^{N}, &{}\quad \text {if }K_{n}=B_{n},C_{n},D_{n}. \end{array} \right. \end{aligned}$$

We give the condensed.

### Theorem 3.17

Let $f\in \Omega ,$ $0<\beta <1,$ $x\in Y;$ *n*,  $\mu >0;$ $N\in {\mathbb {N}}$ with $n^{1-\beta }>2.$ Then

(i) 94 $$\begin{aligned} \left\| K_{n}\left( f,x\right) -f\left( x\right) \right\| _{\gamma }\le c_{N}\left[ \omega _{1}\left( f,\Lambda \left( n\right) \right) +\frac{ 2\left\| \left\| f\right\| _{\gamma }\right\| _{\infty }}{{\textrm{e}}^{\mu \left( n^{1-\beta }-2\right) }}\right] =:\tau \left( n\right) , \end{aligned}$$
  where $\omega _{1}$ is for $p=\infty ,$
  and
(ii) 95 $$\begin{aligned} \left\| \left\| K_{n}\left( f\right) -f\right\| _{\gamma }\right\| _{\infty }\le \tau \left( n\right) \rightarrow 0, \quad \text {as } n\rightarrow \infty . \end{aligned}$$

For *f* uniformly continuous and in $\Omega ,$ we obtain

$$\begin{aligned} \underset{n\rightarrow \infty }{\lim }K_{n}\left( f\right) =f, \end{aligned}$$

pointwise and uniformly.

### Proof

By Theorems 3.1, 3.10, 3.11 and 3.12. $\square$

Next, we do iterated neural network approximation (see also [10]).

We make the following.

### Remark 3.18

Let $r\in {\mathbb {N}}$ and $K_{n}$ as above. We observe that

$$\begin{aligned}{} & {} K_{n}^{r}f-f=\left( K_{n}^{r}f-K_{n}^{r-1}f\right) +\left( K_{n}^{r-1}f-K_{n}^{r-2}f\right) \\{} & {} \quad + \left( K_{n}^{r-2}f-K_{n}^{r-3}f\right) +\cdots +\left( K_{n}^{2}f-K_{n}f\right) +\left( K_{n}f-f\right) . \end{aligned}$$

Then

$$\begin{aligned}{} & {} \left\| \left\| K_{n}^{r}f-f\right\| _{\gamma }\right\| _{\infty }\le \left\| \left\| K_{n}^{r}f-K_{n}^{r-1}f\right\| _{\gamma }\right\| _{\infty }+\left\| \left\| K_{n}^{r-1}f-K_{n}^{r-2}f\right\| _{\gamma }\right\| _{\infty }\\{} & {} \qquad + \left\| \left\| K_{n}^{r-2}f-K_{n}^{r-3}f\right\| _{\gamma }\right\| _{\infty }+\cdots +\left\| \left\| K_{n}^{2}f-K_{n}f\right\| _{\gamma }\right\| _{\infty }+\left\| \left\| K_{n}f-f\right\| _{\gamma }\right\| _{\infty }\\{} & {} \quad = \left\| \left\| K_{n}^{r-1}\left( K_{n}f-f\right) \right\| _{\gamma }\right\| _{\infty }+\left\| \left\| K_{n}^{r-2}\left( K_{n}f-f\right) \right\| _{\gamma }\right\| _{\infty }+\left\| \left\| K_{n}^{r-3}\left( K_{n}f-f\right) \right\| _{\gamma }\right\| _{\infty }\\{} & {} \qquad +\cdots +\left\| \left\| K_{n}\left( K_{n}f-f\right) \right\| _{\gamma }\right\| _{\infty }+\left\| \left\| K_{n}f-f\right\| _{\gamma }\right\| _{\infty }\le r\left\| \left\| K_{n}f-f\right\| _{\gamma }\right\| _{\infty }. \end{aligned}$$

That is

96 $$\begin{aligned} \left\| \left\| K_{n}^{r}f-f\right\| _{\gamma }\right\| _{\infty }\le r\left\| \left\| K_{n}f-f\right\| _{\gamma }\right\| _{\infty }. \end{aligned}$$

We give the following.

### Theorem 3.19

All here as in Theorem 3.17 and $r\in {\mathbb {N}},$ $\tau \left( n\right)$ as in (94). Then

97 $$\begin{aligned} \left\| \left\| K_{n}^{r}f-f\right\| _{\gamma }\right\| _{\infty }\le r\tau \left( n\right) . \end{aligned}$$

So that the speed of convergence to the unit operator of $K_{n}^{r}$ is not worse than of $K_{n}.$

### Proof

As similar to [18] is omitted. $\square$

### Remark 3.20

Let $$m_{1},m_{2},\ldots ,m_{r}\in {\mathbb {N}}:m_{1}\le m_{2}\le \cdots \le m_{r},$$ $$0<\beta <1,\mu >0,$$ $$f\in \Omega .$$ Then

$$\begin{aligned} \Lambda \left( m_{1}\right) \ge \Lambda \left( m_{2}\right) \ge \cdots \ge \Lambda \left( m_{r}\right) ,\quad \Lambda \text { as in } (91). \end{aligned}$$

Therefore

$$\begin{aligned} \omega _{1}\left( f,\Lambda \left( m_{1}\right) \right) \ge \omega _{1}\left( f,\Lambda \left( m_{2}\right) \right) \ge \cdots \ge \omega _{1}\left( f,\Lambda \left( m_{r}\right) \right) . \end{aligned}$$

Assıme further that $m_{i}^{1-\beta }>2,$ $i=1,\ldots ,r.$ Then

$$\begin{aligned} \frac{1}{{\textrm{e}}^{\mu \left( m_{1}^{1-\beta }-2\right) }}\ge \frac{1}{{\textrm{e}}^{\mu \left( m_{2}^{1-\beta }-2\right) }}\ge \cdots \ge \frac{1}{{\textrm{e}}^{\mu \left( m_{r}^{1-\beta }-2\right) }}. \end{aligned}$$

Let $K_{m_{i}}$ as above, $i=1,\ldots ,r,$ all of the same kind. We write

$$\begin{aligned}{} & {} K_{m_{r}}\left( K_{m_{r-1}}\left( \ldots K_{m_{2}}\left( K_{m_{1}f}\right) \right) \right) -f\\{} & {} \quad = K_{m_{r}}\left( K_{m_{r-1}}\left( \ldots K_{m_{2}}\left( K_{m_{1}f}\right) \right) \right) -K_{m_{r}}\left( K_{m_{r-1}}\left( \ldots K_{m_{2}}f\right) \right) \\{} & {} \qquad +K_{m_{r}}\left( K_{m_{r-1}}\left( \ldots K_{m_{2}}f\right) \right) -K_{m_{r}}\left( K_{m_{r-1}}\left( \ldots K_{m_{3}}f\right) \right) \\{} & {} \qquad +K_{m_{r}}\left( K_{m_{r-1}}\left( \ldots K_{m3}f\right) \right) -K_{m_{r}}\left( K_{m_{r-1}}\left( \ldots K_{m_{4}}f\right) \right) \\{} & {} \qquad +\cdots + K_{m_{r}}\left( K_{m_{r-1}}f\right) -K_{m_{r}}f+K_{m_{r}}f-f\\{} & {} \quad = K_{m_{r}}\left( K_{m_{r-1}}\left( \ldots K_{m_{2}}\right) \right) \left( K_{m_{1}}f-f\right) +K_{m_{r}}\left( K_{m_{r-1}}\left( \ldots K_{m_{3}}\right) \right) \left( K_{m_{2}}f-f\right) \\{} & {} \qquad +K_{m_{r}}\left( K_{m_{r-1}}\left( \ldots K_{m_{4}}\right) \right) \left( K_{m3}f-f\right) +\cdots +K_{m_{r}}\left( K_{m_{r-1}}f-f\right) +K_{m_{r}}f-f. \end{aligned}$$

Hence, by the triangle inequality of $\left\| \left\| \cdot \right\| _{\gamma }\right\| _{\infty },$ we get

$$\begin{aligned}{} & {} \left\| \left\| K_{m_{r}}\left( K_{m_{r-1}}\left( \ldots K_{m_{2}}\left( K_{m_{1}}f\right) \right) \right) -f\right\| _{\gamma }\right\| _{\infty }\\{} & {} \quad \le \left\| \left\| K_{m_{r}}K_{m_{r-1}}\ldots K_{m_{2}}\left( K_{m_{1}}f-f\right) \right\| _{\gamma }\right\| _{\infty }\\{} & {} \qquad +\left\| \left\| K_{m_{r}}K_{m_{r-1}}\ldots K_{m_{2}}\left( K_{m_{1}}f-f\right) \right\| _{\gamma }\right\| _{\infty }\\{} & {} \qquad +\left\| \left\| K_{m_{r}}\left( K_{m_{r-1}}\left( \ldots K_{m_{4}}\right) \right) \left( K_{m_{3}}f-f\right) \right\| _{\gamma }\right\| _{\infty }\\{} & {} \qquad +\cdots +\left\| \left\| K_{m_{r}}\left( K_{m_{r-1}}f-f\right) \right\| _{\gamma }\right\| _{\infty }+\left\| \left\| K_{m_{r}}f-f\right\| _{\gamma }\right\| _{\infty }\le \end{aligned}$$

[repeatedly applying (87)]

$$\begin{aligned}{} & {} \left\| \left\| K_{m_{_{1}}}f-f\right\| _{\gamma }\right\| _{\infty }+\left\| \left\| K_{m_{_{2}}}f-f\right\| _{\gamma }\right\| _{\infty }+\left\| \left\| K_{m_{_{3}}}f-f\right\| _{\gamma }\right\| _{\infty }\\{} & {} \quad +\cdots + \left\| \left\| K_{m_{_{r-1}}}f-f\right\| _{\gamma }\right\| _{\infty }+\left\| \left\| K_{m_{2}}f-f\right\| _{\gamma }\right\| _{\infty }+\left\| \left\| K_{m_{3}}f-f\right\| _{\gamma }\right\| _{\infty }\\{} & {} \quad +\cdots + \left\| \left\| K_{m_{_{r-1}}}f-f\right\| _{\gamma }\right\| _{\infty }+\left\| \left\| K_{m_{r}}f-f\right\| _{\gamma }\right\| _{\infty }=\sum _{i=1}^{r}\left\| \left\| K_{m_{i}}f-f\right\| _{\gamma }\right\| _{\infty }. \end{aligned}$$

That is, we proved

98 $$\begin{aligned} \left\| \left\| K_{m_{r}}\left( K_{m_{r-1}}\left( \ldots K_{m_{2}}\left( K_{m_{1}}f\right) \right) \right) -f\right\| _{\gamma }\right\| _{\infty }\le \sum _{i=1}^{r}\left\| \left\| K_{m_{i}}f-f\right\| _{\gamma }\right\| _{\infty }. \end{aligned}$$

We also present the following.

### Theorem 3.21

Let $f\in \Omega ;$ *m*,  *N*,  $m_{1},m_{2},\ldots ,m_{r}\in {\mathbb {N}}:m_{1}\le m_{2}\le \cdots \le m_{r},$ $0<\beta <1,\mu >0;$ $m_{i}^{1-\beta }>2,$ $i=1,\ldots ,r,$ $x\in Y,$ and let $\left( K_{m_{1}},\ldots ,K_{m_{r}}\right)$ as $\left( L_{m_{1}},\ldots ,L_{m_{r}}\right)$ or $\left( B_{m_{1}},\ldots ,B_{m_{r}}\right)$ or $\left( C_{m_{1}},\ldots ,C_{m_{r}}\right)$ or $\left( D_{m_{1}},\ldots ,D_{m_{r}}\right) ,$ $p=\infty .$ Then

99 $$\begin{aligned}{} & {} \left\| K_{m_{r}}\left( K_{m_{r-1}}\left( \ldots K_{m_{2}}\left( K_{m_{1}}f\right) \right) \right) \left( x\right) -f\left( x\right) \right\| _{\gamma }\nonumber \\{} & {} \quad \le \left\| \left\| K_{m_{r}}\left( K_{m_{r-1}}\left( \ldots K_{m_{2}}\left( K_{m_{1}}f\right) \right) \right) -f\right\| _{\gamma }\right\| _{\infty }\nonumber \\{} & {} \quad \le \sum _{i=1}^{r}\left\| \left\| K_{m_{i}}f-f\right\| _{\gamma }\right\| _{\infty }\nonumber \\{} & {} \quad \le c_{N}\sum _{i=1}^{r}\left[ \omega _{1}\left( f,\Lambda \left( m_{i}\right) \right) +\frac{2\left\| \left\| f\right\| _{\gamma }\right\| _{\infty }}{{\textrm{e}}^{\mu \left( m_{i}^{1-\beta }-2\right) }}\right] \nonumber \\{} & {} \quad \le rc_{N}\left[ \omega _{1}\left( f,\Lambda \left( m_{1}\right) \right) +\frac{ 2\left\| \left\| f\right\| _{\gamma }\right\| _{\infty }}{{\textrm{e}}^{\mu \left( m_{1}^{1-\beta }-2\right) }}\right] . \end{aligned}$$

Clearly,  we notice that the speed of convergence to the unit operator of the multiply iterated operator is not worse than the speed of $K_{m_{1}}.$

### Proof

As similar to [18] is omitted. $\square$

We continue with the following.

### Theorem 3.22

Let all as in Corollary 3.9, and $r\in {\mathbb {N}}.$ Here,  $\Lambda _{3}\left( n\right)$ is as in (73). Then

100 $$\begin{aligned} \left\| \left\| L_{n}^{r}f-f\right\| _{\gamma }\right\| _{\infty }\le r\left\| \left\| L_{n}f-f\right\| _{\gamma }\right\| _{\infty }\le r\Lambda _{3}\left( n\right) . \end{aligned}$$

### Proof

As similar to [18] is omitted. $\square$

Next, we present some $L_{p_{1}},$ $p_{1}\ge 1,$ approximation related results.

### Theorem 3.23

Let $p_{1}\ge 1,$ $f\in C\left( \prod _{i=1}^{n}\left[ a_{i},b_{i}\right] ,X\right) ,$ $0<\beta <1,\mu >0;$ $N,n\in {\mathbb {N}}$ with $n^{1-\beta }>2,$ and $\lambda _{1}\left( n\right)$ as in (38), $\omega _{1}$ is for $p=\infty .$ Then

101 $$\begin{aligned} \left\| \left\| L_{n}f-f\right\| _{\gamma }\right\| _{p_{1},\prod _{i=1}^{n}\left[ a_{i},b_{i}\right] }\le \lambda _{1}\left( n\right) \left( \prod _{i=1}^{n}\left( b_{i}-a_{i}\right) \right) ^{\frac{1}{p_{1}}}. \end{aligned}$$

We notice that $\lim _{n\rightarrow \infty } \left\| \left\| L_{n}f-f\right\| _{\gamma }\right\| _{p_{1},\prod _{i=1}^{n} \left[ a_{i},b_{i}\right] }=0.$

### Proof

Obvious, by integrating (38), etc. $\square$

It follows:

### Theorem 3.24

Let $p_{1}\ge 1,$ $f\in C_{B}\left( {\mathbb {R}}^{N},X\right) ,$ $0<\beta <1,\mu >0;$ $N,n\in {\mathbb {N}}$ with $n^{1-\beta }>2,$ and $\omega _{1}$ is for $p=\infty ;$ $\lambda _{2}\left( n\right)$ as in (74) and *P* a compact set of ${\mathbb {R}}^{N}.$ Then

102 $$\begin{aligned} \left\| \left\| B_{n}f-f\right\| _{\gamma }\right\| _{p_{1}, P}\le \lambda _{2}\left( n\right) \left| P\right| ^{\frac{1 }{p_{1}}}, \end{aligned}$$

where $\left| P\right| <\infty ,$ is the Lebesgue measure of *P*. We notice that $\lim _{n\rightarrow \infty }\left\| \left\| B_{n}f-f\right\| _{\gamma }\right\| _{p_{1},P}=0$ for $f\in \left( C_{U}\left( {\mathbb {R}}^{N},X\right) \cap C_{B}\left( {\mathbb {R}}^{N},X\right) \right) .$

### Proof

By integrating (74), etc. $\square$

Next come.

### Theorem 3.25

All as in Theorem 3.24, but we use $\lambda _{3}\left( n\right)$ of (78). Then

103 $$\begin{aligned} \left\| \left\| C_{n}f-f\right\| _{\gamma }\right\| _{p_{1}, P}\le \lambda _{3}\left( n\right) \left| P\right| ^{\frac{1 }{p_{1}}}. \end{aligned}$$

We have that $\lim _{n\rightarrow \infty } \left\| \left\| C_{n}f-f\right\| _{\gamma }\right\| _{p_{1}, P}=0$ for $f\in \left( C_{U}\left( {\mathbb {R}}^{N},X\right) \cap C_{B}\left( {\mathbb {R}} ^{N},X\right) \right) .$

### Proof

By (78). $$\square $$

### Theorem 3.26

All as in Theorem 3.24, but we use $\lambda _{4}\left( n\right)$ of (84). Then

104 $$\begin{aligned} \left\| \left\| D_{n}f-f\right\| _{\gamma }\right\| _{p_{1}, P}\le \lambda _{4}\left( n\right) \left| P\right| ^{\frac{1 }{p_{1}}}. \end{aligned}$$

We have that $\lim _{n\rightarrow \infty } \left\| \left\| D_{n}f-f\right\| _{\gamma }\right\| _{p_{1}, P}=0$ for $f\in \left( C_{U}\left( {\mathbb {R}}^{N},X\right) \cap C_{B}\left( {\mathbb {R}} ^{N},X\right) \right) .$

### Proof

By (84). $$\square $$

### Application 3.27

A typical application of all of our results is when $\left( X,\left\| \cdot \right\| _{\gamma }\right) =\left( {\mathbb {C}},\left| \cdot \right| \right) ,$ where ${\mathbb {C}}$ is the set of the complex numbers.

## Data Availability

Not applicable.
